# Evolution of Toll-like receptor 7/8 agonist therapeutics and their delivery approaches: From antiviral formulations to vaccine adjuvants

**DOI:** 10.1016/j.addr.2021.05.013

**Published:** 2021-05-29

**Authors:** Sachin Bhagchandani, Jeremiah Johnson, Darrell J. Irvine

**Affiliations:** 1 Koch Institute for Integrative Cancer Research, Massachusetts Institute of Technology, Cambridge, MA 02139 USA.; 2 Department of Biological Engineering, Massachusetts Institute of Technology, Cambridge, MA 02139 USA.; 3 Department of Materials Science and Engineering, Massachusetts Institute of Technology, Cambridge, MA 02139 USA.; 4 Department of Chemistry, Massachusetts Institute of Technology, Cambridge, MA 02139 USA.; 5 Department of Chemical Engineering, Massachusetts Institute of Technology, Cambridge, MA 02139 USA.; 6 Broad Institute of MIT and Harvard, Cambridge, MA 02139 USA.; 7 Howard Hughes Medical Institute, Chevy Chase, MD 20815 USA.; 8 Ragon Institute of MGH, MIT, and Harvard, Cambridge, MA 02139 USA.

**Keywords:** Toll-like receptors, TLR7 agonists, immunomodulators, cancer, infectious disease, bioconjugation, vaccine adjuvants, nanoparticles

## Abstract

Imidazoquinoline derivatives (IMDs) and related compounds function as synthetic agonists of Toll-like receptors 7 and 8 (TLR7/8) and one is FDA approved for topical antiviral and skin cancer treatments. Nevertheless, these innate immune system-activating drugs have potentially much broader therapeutic utility; they have been pursued as antitumor immunomodulatory agents and more recently as candidate vaccine adjuvants for cancer and infectious disease. The broad expression profiles of TLR7/8, poor pharmacokinetic properties of IMDs, and toxicities associated with systemic administration, however, are formidable barriers to successful clinical translation. Herein, we review IMD formulations that have advanced to the clinic and discuss issues related to biodistribution and toxicity that have hampered the further development of these compounds. Recent strategies aimed at enhancing safety and efficacy, particularly through the use of bioconjugates and nanoparticle formulations that alter pharmacokinetics, biodistribution, and cellular targeting, are described. Finally, key aspects of the biology of TLR7 signaling, such as TLR7 tolerance, that may need to be considered in the development of new IMD therapeutics are discussed.

## 1. Introduction

**Figure 1.**
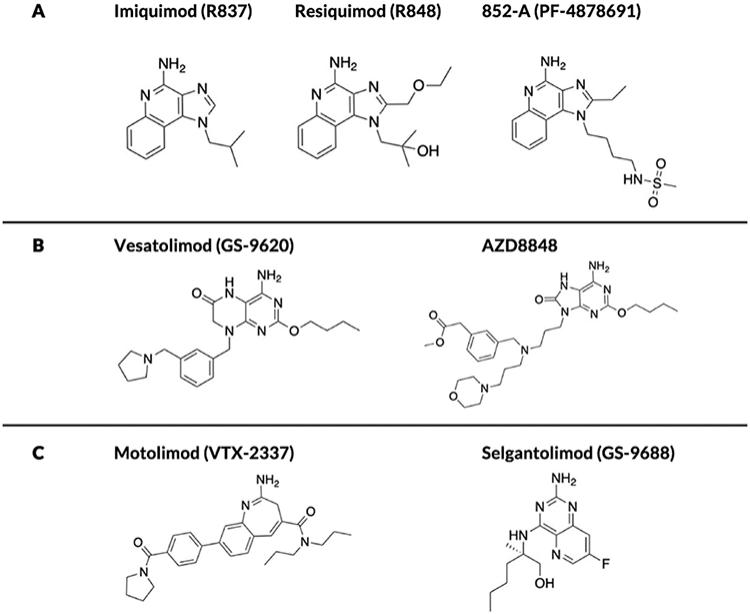
Representative structures of widely used synthetic TLR7/8 agonists. (A) Imidazoquinoline derivatives (IMDs) (B) pteridinone-based (GS-9620) and 8-oxoadenine (AZD-8848) derivatives (C) TLR8-specific benzazepine (VTX-2337) and pyrimidine (GS-9688) analogues.

In the mid-1980s, 3M Pharmaceuticals discovered that imidazoquinoline derivatives (IMDs) could possess antiviral properties [1]. At that time, the functions of Toll-like receptors (TLRs) were not defined and the antiviral feature of IMDs was not known to be related to TLR7/8 activation [2]. The first patent filing on this class of compounds focused on topical administration and detailed their efficacy in a guinea pig model of herpes simplex virus [3]. One of the first IMDs reported, imiquimod (also known as R837; Fig. 1A), progressed through clinical trials and was approved by the United States Food and Drug Administration (FDA) in 1997 for the topical treatment of genital and perianal warts and actinic keratosis (Aldara, 5%). It has since been approved for topical treatment of basal cell carcinoma [4]. Following the success of R837, a library of more potent and selective IMDs was developed and tested in preclinical studies and clinical trials [5,6]. A prominent compound in this class is resiquimod (R848; Fig. 1A), which was shown to be 100 times more potent than R837 in stimulating TLR7 [7]. Nevertheless, R848 has followed a more difficult path to the clinic, with multiple human trials in genital herpes and hepatitis C yielding no positive outcomes [8]. These disappointing results led 3M to discontinue development of R848 and license it to Spirig Pharma, which conducted clinical trials testing R848 on basal cell carcinoma and actinic keratosis without success [9]. Spirig Pharma was acquired by Galderma, and R848 has since received orphan status from the European Medicines Agency and is in continued clinical development for the treatment of cutaneous T cell lymphoma [10].

The transition from treating viral infections to malignant tumors using IMDs was bolstered by several mechanistic insights into the biology of these molecules as activators of TLR7/8 [11]. In addition to inducing an antiviral state through secretion of type I interferons (IFNs), synthetic TLR7/8 agonists trigger acute inflammation and stimulate adaptive immunity by activating the nuclear factor kappa-light-chain enhancer of activated B cells (NF-κB) pathway [12]. This mechanistic rationale led compounds such as 852-A (Fig. 1A) to be tested for treatment of hematological malignancies (e.g., chronic lymphocytic leukemia) and solid tumors (e.g., ovarian, cervical, and breast cancers) [13]. In fact, 852-A and R848 generated sufficient excitement that, in 2008, they were both featured on the National Cancer Institute’s list of 20 agents with the highest potential for treating cancer [14]. Nevertheless, while potent immune activation was a consistent feature of the 852-A and R848 trials, severe adverse events associated with on-target, off-tumor activity hindered attempts to take these compounds from bench to bedside [15]. In parallel, mechanistic insights into the impact of TLR signaling on T cell immunity prompted studies focused on the use of IMDs as allergy and asthma treatments with the goal of skewing pathological Th2 immune responses toward a non-pathologic Th1 functional profile [16]. Promising results were obtained in large animal models and preliminary clinical work, but the toxicity of IMDs once again dampened success and forced the field to address systemic side effects using approaches rooted in medicinal chemistry, formulation, prodrug design, and drug delivery [17].

Despite the challenges described above, there is maintained clinical interest in the development of IMDs and related synthetic immune agonists, which is driven by significant progress in understanding innate immune sensors and their link to the adaptive immune system [18]. Crystal structures of TLR8 and TLR7 both with and without ligands have recently been elucidated, enabling structure-based compound development aimed toward enhanced receptor specificity [19]. These advances are especially important because R837 has been shown to have immune effects independent of TLR7/8, which could hamper attempts to reduce its off-target toxicity [20]. Today, in response to the multitude of toxicity-induced failures, research focus is moving toward localized delivery approaches, especially in the context of cancer, and prodrug or delivery formulations aiming to target IMDs to desired tissues and/or cells [21]. Here, we review preclinical and clinical studies of IMDs and related compounds, highlighting new strategies to overcome the safety challenges and address early clinical failures.

## 2. TLR7/8 biology and cellular implications of downstream effects

Pioneering work by Akira and others laid the foundation of TLR7/8 biology, and demonstrated the structural basis for recognition of guanosine- and uridine-rich viral single-stranded RNA (the likely natural ligands for these TLRs) [22-24]. We provide here a brief overview of the currently known signaling pathways and immune effector modules downstream of TLR7/8 agonists that will allow for an appreciation of the complexities involved and highlight the important players—the cells and proteins in the immune cascade—that will be discussed in later sections focused on preclinical and clinical studies.

### 2.1 TLR7/8 signaling pathways & downstream effects

**Figure 2.**
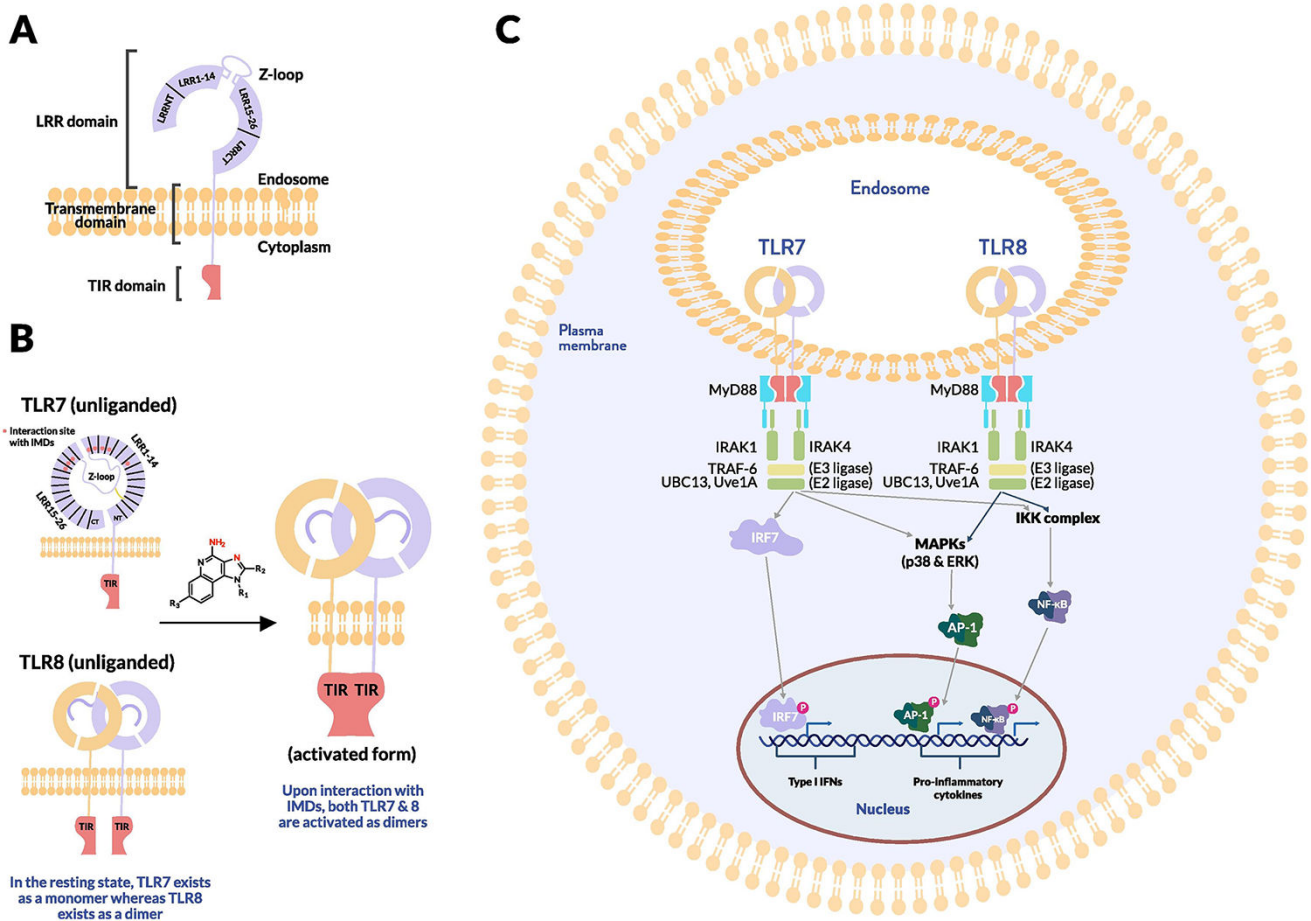
TLR7/8 signaling pathways and downstream immune-effector modules. (A) Representative structure of TLR7/8 receptor. (B) Schematic of TLR7/8 receptor in the resting and activated states. (C) TLR7/8 signaling pathways from dimerization of the receptors to activation of the 3 transcriptional factors i.e., AP-1, NF-κB and IRF7.

The TLRs are a family of 13 type 1 transmembrane proteins expressed both by tissue cells and immune cells that function as pattern recognition receptors (PRRs); that is, they identify conserved molecular structures on microbes or molecules released by dying cells and thereby comprise a major form of innate immune sensing [25]. TLRs share a common general structure: The N-terminal ectodomain consists of leucine-rich repeats (LRRs), formed in a horseshoe shape, followed by a single transmembrane domain and a cytosolic Toll-interleukin-1 receptor (TIR) domain [26] (Fig. 2A). TLRs dimerize upon binding their cognate ligand (Fig. 2B), leading to conformational changes that allow for the recruitment of adapter molecules and initiating signaling cascades that ultimately induce transcription of inflammatory mediators [27]. In the case of TLR7/8, dimerization initiates the TIR signaling cascade, which results in association with the adaptor protein myeloid differentiation primary response 88 (MyD88) at its carboxy terminus [28]. MyD88 also has an N-terminal death domain that recruits and associates with similar death domains present on two serine-threonine protein kinases: IL-1-receptor associated kinase (IRAK4) and IRAK1 [29] (Fig. 2C).

The above complex consisting of MyD88, IRAK4, and IRAK1 recruits the enzyme tumor necrosis factor (TNF) receptor-associated factor 6 (TRAF6) [30]. TRAF-6 is an E3 ubiquitin ligase that, in combination with UBC13 (an E2 ubiquitin ligase) and Uve1A (a cofactor), initiates polymerization of ubiquitin via K63 linkages [31]. This polymerized scaffold recruits a signaling complex, resulting in activation of the AP-1 family transcription factors that transcribe cytokine genes such as TNF-α. The same scaffold also leads to the phosphorylation of the inhibitor of κB (IκB), the protein responsible for keeping NF-κB in the cytoplasm. This action results in the degradation of IκB, which releases NF-κB into the nucleus where it drives transcription of genes encoding proinflammatory cytokines such as IL-6 and TNF-α, resulting in a state of acute inflammation [32] (Fig. 2C).

In addition, IRAK1, as part of the IRAK complex, can physically associate with interferon regulatory factor 7 (IRF7), which is highly expressed in specific cell subsets such as plasmacytoid dendritic cells [33]. Phosphorylated IRF7 enters the nucleus to induce expression of type I IFNs, inducing an antiviral state (Fig. 2C).

### 2.2 Cell types responding to TLR7/8 ligands

Although the immune-effector modules of TLR7/8 activation can be broadly classified into “acute inflammation” and “antiviral state,” the downstream effects of TLR7/8 activation vary by cell type. Table 1 summarizes the current understanding of TLR7/8 expression in different cells across species [34-37]. Defining TLR expression patterns has been confounded by a lack of reliable antibodies for TLRs, conflicting results with different primer sequences used in reverse transcription (RT)-PCR measurements, and species-specific differences in TLR7/8 expression across cell types. Additionally, similar cell types might express different levels of TLR7/8 in different tissues, and this pattern of expression can be further altered by the activation, maturation or differentiation of the cells [38]. Under certain circumstances, expression may be induced even in cells with low basal TLR7 levels, in particular immune cells such as macrophages and monocytes [39]. Most studies have focused on quantifying TLR7/8 expression in immune cells and less is known about expression levels in other cell subsets, such as epithelial, endothelial or hematopoietic cells.

Dendritic cells (DCs) are broadly classified into conventional/myeloid DCs (cDCs), which express CD11c, or plasmacytoid DCs (pDCs), which do not express CD11c [40]. In humans, cDCs express TLR8 mRNA, but their expression of TLR7 is still under debate [41]. In mice, cDCs can be further subdivided into CD4^+^, CD8^+^, or double negative cells, of which only CD8α^+^ DCs lack TLR7 expression and fail to respond to synthetic TLR7 ligands [42]. pDCs, unlike cDCs, express high levels of TLR7 mRNA but lack TLR8 expression [43].

Upon TLR7/8 ligation in the presence of antigens, cDCs undergo multiple functional changes, including maturation and expression of co-stimulatory markers, such as CD80 and CD86, and secretion of proinflammatory cytokines, including IL-12, TNF-α, and IL-6 [44]. The migration of DCs to draining lymph nodes (dLNs) to stimulate naïve T cells is triggered by TLR7/8-induced downregulation of inflammatory chemokine receptors such as CCR6 in concert with upregulation of receptors for lymphoid chemokines such as CCR7 [45]. Activation of naïve T cells by DCs is contingent on receiving three signals: the first is provided by binding of the T cell receptor (TCR) to antigenic peptide presented by the major histocompatibility complexes (MHCs) present on DCs; the second signal is provided by co-stimulatory molecules such as CD80, CD86, or CD40, which trigger CD28 expression on naïve T cells; and the third signal is provided by cytokines, especially IL-12, which have further downstream effects on multiple immune subsets [46]. For example, increased IL-12 levels lead to IFN-γ secretion by T cells and natural killer (NK) cells, further amplifying the immunological cascade [47-49]. Thus, T cell activation depends upon the class of MHC that presents the antigenic peptide, the co-stimulatory environment, and the cytokine milieu [50].

Type 1 IFNs are one of the key products of TLR7/8 activation. In both humans and mice, upon TLR7 ligation, pDCs secrete large amounts of type I IFNs (IFN-α and IFN-β) [51]. Although ligands for other innate immune receptors such as TLR9 and more recently the STimulator of Interferon Genes (STING) have been explored for their capacity to induce type I IFNs [52,53], targeting TLR7/8 with synthetic agonist drugs may have some advantages over these other innate immune receptors. For example, the substantial difference in cellular distribution of TLR9 between rodents and humans and the rapid degradation of the most common cyclic-dinucleotide STING agonists by circulating and cell-bound enzymes are but a few of the limitations that widen the appeal of TLR7/8 agonists as potent immune modulators. The downstream effects of this type 1 IFN production are discussed in disease-specific contexts in Section 3.

Beyond DCs (arguably the most important cell type for TLR7/8 agonists), other innate immune cells such as monocytes, macrophages, neutrophils, mast cells, and eosinophils also express TLR7/8 and secrete a host of cytokines and chemokines upon ligation by synthetic agonists, as shown in Table 1 [54-63].

One thought-provoking aspect of the cellular effects of TLR7/8 ligation is the duality of direct and indirect immune processes [64]. Jakubzick and coworkers demonstrated that, in the mouse lung microenvironment, CD11b^+^ DCs need to be directly activated by TLR7 to efficiently cross-present antigens, whereas CD103^+^ (CD8α^+^) DCs require TLR3 activation [65]. This observation further supports theories by Reis e Sousa and colleagues on the requirement for direct DC activation to efficiently prime T cell activity [66]. Multiple studies, however, have indicated a role for indirect DC activation in priming the adaptive immune system. For example, type I IFN secreted from pDCs has been shown to activate cross-presenting CD8α^+^ DCs [64,67]. Apart from cellular immunity, B cells also express TLR7; ligation by synthetic TLR7 agonists has been shown to enhance activation of multiple B cell subsets [68-73].

### 2.3. Structure-activity relationships of IMD TLR7/8 agonists

**Figure 3.**
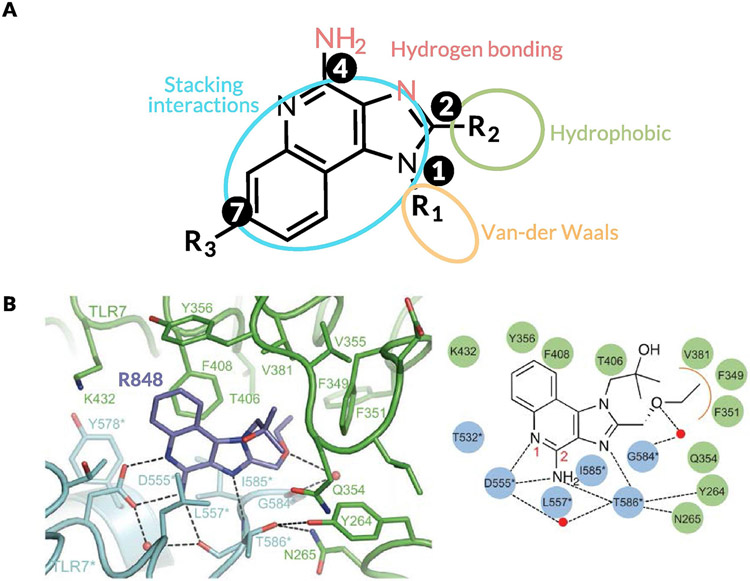
SAR studies on synthetic TLR7/8 agonists. (A) Structure of IMDs indicating the sites at which substitutions were studied for improving potency. (B) Crystal structure of TLR7 bound to synthetic TLR7/8 agonist R848 highlighting the important interactions between the receptor and synthetic ligand.

The biological effects of these TLR7/8 agonists are heavily dependent on their chemical structures. Most of the structure–activity relationship (SAR) studies on IMDs were performed before the crystal structures of liganded TLR8 and TLR7 were elucidated in 2013 and 2016, respectively [74,75]. Moreover, in early SAR studies, Gerster and colleagues measured *in vitro* production of IFN-α in human peripheral blood mononuclear cells (hPBMCs) as a surrogate biomarker for potency (TLR7-specific bioassays were not available at the time) [76]. Nevertheless, important insights were gained from these earlier studies. As shown in Fig. 3A, the major sites for derivatization of the IMD core have been the N-1, C-2, C-4 amine, and the C-7 position. These studies showed that attachment of long alkyl chains or bulky substituents at N-1 or C-2 reduced IFN production, whereas short-chain alkyl substituents at C-2 and short hydroxyl chains at N-1 enhanced IFN production. Most importantly, the C-4 amine was shown to be a key requirement for activity; all substitutions at this position completely abrogated IFN-α production. Moreover, most substitutions on the aryl ring were not tolerated except at the C-7 position. David and colleagues synthesized a library of more than 50 compounds to further demonstrate the increased potency obtained via substituents at N-1 and C-2 using both IFN-α production from hPBMCs and the current standard HEK-TLR7/8 reporter gene assay [77]. The lead compounds were shown to be exceptionally potent inducers of cytokine production in human newborn and adult leukocytes, and the crystal structures generated for the liganded TLRs validated the positive effects of the N-1 and C-2 substituents [78]. Ferguson and colleagues showed improved potency with C-7 methoxy carbonyl derivatives, hypothesizing that this result could be due to inflammasome activation [79]. The same group further studied the effects of C-2 and N-1 substituents on the C-7 substituted scaffold and showed that TLR8 activity correlated with a hydrogen bond donor at the N-1 position, with compounds that contained a terminal amino group showing the highest potency [80,81]. These studies followed work by David and colleagues that showed quinoline amine-based derivatives lacking the imidazole groups were more potent and selective TLR8 agonists, with the terminal amino substituent extending into a pocket within the TLR8 ectodomain, forming hydrogen bonds with glycine 351 [82]. The same group explored IMD-based dimers (linked at N-1), trimers, and hexamers with improved potency that were validated *in vivo* in vaccine studies [83,84]. Extensive SAR studies on imidazopyridines, thiazoquinolines, furopyridines, and furoquinolines have been conducted by the David group in search of potent and specific TLR7 and TLR8 agonists [85-88].

The elucidation of the crystal structures of liganded TLR8 and TLR7 by Shimizu and colleagues, and the resulting identification of key interactions between the ligands and the receptors, has paved the way for the more targeted design of potent TLR7/8 agonists (Fig. 3B) [89]. For TLR7, the effects of N-1 substituents depended on van der Waals contacts with the loop region of LRR11 [90]. The pocket formed by phenylalanine 349, phenylalanine 351, valine 381, and phenylalanine 408 interacts with C-2 substituents via hydrophobic interactions (Fig. 3B). This interaction was particularly essential for TLR7 activity because R837 lacks the C-2 substituent and demonstrates weak TLR7 agonism. Similarly, for TLR8 agonism, stacking interactions or hydrogen bonds with phenylalanine 405 and aspartic acid 543 are critical components that need to be conserved in the ligand structure [91]. These insights have led to the development of synthetic ligands with higher potency and represent a key milestone on the path to TLR7/8 agonism for improved therapeutic outcomes [92,93].

Although we focus here on SAR studies of IMDs because they are the most studied, other classes of synthetic TLR7/8 agonists that expand upon nucleobase and ribonucleoside analogs include adenine-, guanosine-, and pteridinone-based derivatives in addition to benzazepines, benzonaphthyridines, and pyrrolopyrimidines [94-98]. Each of these series of agonists, particularly 8-oxo-adenine derivatives, have been expanded significantly via SAR studies to generate lead compounds that are in clinical trials [99-105].

## 3. Preclinical and Clinical Development of TLR7/8 agonists

In this section, we review preclinical and clinical studies using synthetic TLR7/8 agonists and focus on the clinical results, exploring in detail some of the unexpected outcomes and the hypotheses underlying these responses. Summaries of all of the compounds discussed, organized in order of progress toward clinical approval, are provided in Tables 2 and 3. Additionally, the compounds are classified by application into four areas: (1) skin conditions, the most widespread, with the FDA-approved imiquimod (R837) now having multiple generic formulations [106]; (2) advanced cancers, wherein these molecules serve as tumor immunomodulators and adjuvants; (3) infectious diseases, as antiviral agents, and vaccine adjuvants; and (4) respiratory ailments, as bronchodilators and anti-inflammatories.

### 3.1 Topical therapies for skin conditions

Topical administration of small molecule TLR7/8 agonists formulated as creams or gels overcomes the issues of poor circulation half-life and systemic toxicity that limit the systemic administration of these compounds [107]. Upon topical administration, TLR7/8 agonists induce significant accumulation of pDCs at the treatment site [108]. As discussed previously, pDCs secrete large amounts of type I IFNs in response to TLR7 activation, setting in motion a process that creates an antiviral state in the tissue. The levels of type I IFN-inducible products such as 2'-5'-oligoadenylate synthase (2'-5'-AS) and Mx proteins are increased in the affected area [109]. These products play a crucial role in inhibiting viral replication and survival. For example, 2'-5'-AS is an antiviral enzyme that counteracts viral attack by degrading viral RNA [110]. In addition, TLR7/8 activation causes Langerhans cells (LCs), a type of DCs resident in the epidermis, to differentiate into mature APCs, resulting in the generation of antigen-specific T cells [111]. Finally, the release of proinflammatory cytokines, such as IL-6 and TNF-α, upon NF-κB activation lead to increased NK cell activity and macrophage activation that furthers cytokine release, nitric oxide secretion, and B cell proliferation [112].

The first clinical application of synthetic TLR7/8 agonists was for the treatment of human papillomavirus (HPV)-associated genital and perianal warts. The standard treatment—injection of IFN-α—was expensive but also short-lived, associated with multiple side effects, and mostly unable to prevent recurrence [113]. These warts result from proliferation of keratinocytes infected with HPV and are difficult to treat because they activate basal keratinocytes without activating LCs, the latter of which are necessary for the generation of virus-specific T cells [114]. Topical administration of R837 (imiquimod) was shown to activate LCs, which migrate to the lymph nodes and prime HPV-specific T cells with both cytotoxic and memory phenotypes [115]. Two pivotal phase III studies showed that patients treated with R837 had over 75% reduction in wart area [116,117]. Side effects were minimal, with the most common being erythema at the application site, which correlated with mRNA expression of TNF-α, IFN-γ, and MCP-1. Skin biopsies showed decreases in CD1a mRNA in LCs, indicating activation and migration, as well as upregulation of IFN-γ, IL-2, IL-12p40, CD4, and CD8 mRNA in R837-treated patients. Wart regression was correlated with a decrease in viral load, as shown by decreases in HPV DNA and HPV E7 and L1 mRNA. Based on these results, R837 received FDA approval in 1997 for the treatment of genital and perianal warts.

Another promising, but ultimately unsuccessful, clinical application of R837 and R848 was in the treatment of herpes simplex virus (HSV). There are multiple reports of anecdotal evidence for the successful treatment of both acute and chronic HSV lesions that have resulted in off-label usage of these IMDs [118,119]; however, clinical trials failed due to insufficient efficacy at safe doses (i.e., narrow therapeutic indexes) [120]. Another off-label usage of these synthetic TLR7/8 agonists was for treatment of vaginal or cervical HPV lesions. Multiple clinical trials demonstrated the efficacy of R837 combined with photodynamic therapy or HPV therapeutic vaccines for treatment of vulvar intraepithelial neoplasia (lesions caused by HPV-16) [121,122]. Side effects were significant, however, with one patient needing hospitalization for severe vulval erythema. Moreover, clinical trials in patients with cervical intraepithelial neoplasia have been unsuccessful due to side effects and insufficient enrollment [123].

Beyond virus-induced topical lesions, R837 and R848 have shown promising results in topical treatment of precancerous and cancerous lesions. R837 is FDA-approved for treatment of actinic keratosis, a precancerous skin condition that can develop into squamous cell carcinoma or basal cell carcinoma [124]. In clinical trials, R837 had to compete with photodynamic therapies using 5-aminolevulinic acid as the standard of care [125]. In two phase III trials, R837 showed disease clearance rates greater than 50% (vs. 3% for the control arm) [126]. When the lesions were biopsied, significantly increased levels of CD11c^+^ DCs and CD4^+^ and CD8^+^ T cells were detected.

The third and final indication for which R837 received FDA approval, in 2004, was for basal cell carcinoma, with disease clearance rates of 75% in two phase III trials [127]. Skin biopsies revealed Th1 polarization, significantly increased type I IFN signaling, and recruitment of CXCR3^+^ lymphocytes. Mild erythema, edema, erosion, and ulceration were observed in 50% of patients. Recent clinical results using R848 for treatment of cutaneous T cell lymphoma have also been promising [128,129]. In a phase I trial, 9 of 12 patients showed significant improvement, with 4 experiencing complete clearance of all lesions [130]. The patients who responded to treatment showed significantly increased production of IFN-γ and TNF-α by CD4^+^ T cells, granzyme B by CD8^+^ T cells, and IFN-^γ, perforin, and granzyme by CD56+^ NK cells in skin biopsies of lesions.

For treatment of melanoma, some anecdotal successes using topical administration of IMDs have been reported, but no clinical approvals have been achieved [131]. R837 was studied in clinical trials as the primary treatment for an early form of melanoma called lentigo maligna, but the pathological complete regression rate did not reach the predefined end points needed to replace surgery as the standard treatment [132]. There is anecdotal evidence of topical R837 displaying efficacy in primary and metastatic melanoma with increased levels of CD4^+^ and CD8^+^ T cells in treated skin and sentinel lymph nodes (LNs), as well as regression of skin metastases in breast cancers, but large-scale randomized clinical trials have yet to demonstrate efficacy [133].

Recent clinical studies of IMD topical formulations have been focused on their use as vaccine adjuvants. Over 100 clinical trials using topical imiquimod as a vaccine adjuvant for cancer (mostly DC vaccinations), infectious diseases (hepatitis B vaccination), and respiratory ailments (influenza vaccines) are currently listed on clinicaltrials.gov. These clinical applications are discussed in detail below; it is important to note that despite the success of topical administration of synthetic TLR7/8 agonists, this route of administration has been associated with dose-limiting side effects, such as fever and pemphigus-like lesions, in some cases. These side effects need to be addressed through further molecular design to advance the use of IMDs as vaccine adjuvants [134-136].

### 3.2 IMDs as immunomodulators for Advanced Cancers

In the late 1990s, synthetic TLR7/8 agonists were touted as an alternative to FDA-approved high-dose exogenous IFN-α; widespread use of the latter in treating cancer was limited by significant toxicity and exorbitant cost [137]. Type I IFNs suppress tumor cell proliferation, upregulate expression of MHCs, and increase levels of IFN-γ in synergy with IL-12, thus improving cross-priming by DCs [138]. In certain cancers, synthetic TLR7/8 agonists have been shown to cause apoptosis of tumor cells [139,140]. Other studies, however, show they can promote tumor growth in the lung; thus, the effects of TLR7/8 ligands on different types of tumor cells are cancer and tissue context-dependent [141]. By contrast, the direct and indirect effects of TLR7/8 agonism on immune cells are well defined, with increased infiltration of cytotoxic T cells (via DCs) and NK cells, macrophage polarization, and decreased levels of myeloid-derived suppressor cells combining to yield potent antitumor efficacy [142,143].

The first attempts to demonstrate antitumor efficacy of IMDs studied parenteral administration of 852-A and progressed to phase II clinical trials before discontinuation due to considerable side effects [144,145]. Urosevic and colleagues tested intravenous administration of 852-A in patients with metastatic melanoma [146]. With thrice-weekly dosing, only 4 out of 21 patients showed disease stabilization and 1 patient had a partial response. This lack of efficacy was particularly puzzling given the pharmacologic activity measured; that is, significantly increased levels of type I IFN and activation markers such as CD86 on monocytes in peripheral blood. The authors hypothesized that immune activation might have been decoupled from tumor antigens and that the level of immune activation was insufficient for treating metastatic disease. Other failed attempts at parenteral delivery of TLR agonists for cancer therapy include research on loxoribine, a guanosine analog-based TLR7 agonist. Loxoribine significantly augmented NK cell activity of murine splenocytes, increased cytolytic activity when combined with IL-2 (the synergy was NK cell dependent), and prevented lung metastases in the B16 melanoma mouse model. [147,148]. Nevertheless, when loxoribine was evaluated in a phase I trial in 20 patients with advanced cancers, the disease was stabilized in 6 patients but progressed in 14 patients [149]. Moreover, one patient experienced a 46% decrease in absolute lymphocyte count on day 2 (which returned to baseline by day 8); the trial was discontinued following numerous other immune-related adverse events (irAEs). Preclinical work by Stratford and colleagues on DSR-6434 (a compound from the 8-oxoadenine library discussed in Section 2.3), showed synergy with ionizing radiation in mice bearing CT-26 tumors, with increased levels of IFN-α, IFN-γ, and TNF-α [150]. As a monotherapy, however, DSR-6434 could not elicit effective CD8^+^ T cell responses in animal models. Each of these compounds, although having ultimately failed, provided important insights that have guided current preclinical and clinical developments of synthetic TLR7/8 agonists as antitumor therapies.

Lessons learned from these unsuccessful attempts at parenteral delivery paved the way for next-generation TLR agonists, with a focus on supplementing immune checkpoint inhibitors rather than testing the TLR agonists as monotherapies. Activation of innate immune cells such as DCs by TLR agonists assists in generating potent, tumor-specific immune responses capable of significant tumor regression and long-lasting memory against tumor recurrence [151,152]. Moreover, TLR agonists can directly influence the immunological status of the tumor microenvironment by generating pro-inflammatory cytokines, thus complementing checkpoint inhibitors in overcoming immunosuppressive pathways in tumors [153]. The most clinically advanced TLR agonist in this context is DSP-0509, which is currently in a phase I/II trial in combination with pembrolizumab, the latter of which targets programmed cell death protein 1 (PD-1). Preclinical studies have shown DSP-0509 to have high water solubility, which allows for intravenous administration, resulting in increased serum levels of IFN-α. Antitumor efficacy was demonstrated in CT26 and 4T1 murine tumor models concomitant with upregulation of IFN-γ signature genes, CD8^+^ and effector memory tumor-infiltrating lymphocytes, and MHC class I expression on tumor cells, in addition to decreased levels of myeloid-derived suppressor cells [154]. Systemically administered liposomal formulations of IMDs have also been used in combination with checkpoint blockade therapy. For example, BDB-001, a TLR7/8 agonist formulated in liposomes, is in phase I clinical trials in combination with pembrolizumab. Antibody conjugation has also been explored to enable tumor-targeted IMD delivery. BDC-1001, a TLR7/8 agonist conjugated to the anti-HER2 IgG_1_ monoclonal antibody trastuzumab, is currently in phase I trials in combination with pembrolizumab [155,156]. In the same vein, SBT-6050 is an antibody conjugated TLR8-specific agonist that is advancing toward the clinic based on strong preclinical results. Potent antitumor activity was observed upon intravenous administration in CT26 Her2^+^ tumors, with minimal production of serum cytokines such as IL-6, IL-10, TNF-α, and MCP-1 [157,158]. Thus, parenteral administration of synthetic TLR7/8 agonists continues to receive significant clinical attention, despite failures, because of the potential to treat metastatic disease.

Oral delivery of synthetic TLR7/8 agonists has also been pursued for cancer treatments with the goal of increasing widespread use due to ease of administration and patient compliance. Initial clinical attempts have failed, however, due to insufficient efficacy and unacceptable toxicity. ANA773 (RG-7795) is an orally available TLR7 agonist that was developed by Anadys Pharmaceuticals. Preclinical studies showed promising NK cell-mediated antitumor activity with increased levels of cytokine secretion, cytolysis of tumor cells via increased levels of IFN-γ, and antibody-dependent tumor cytotoxicity mediated by increased levels of IFN-α [159]. Nevertheless, in a phase I clinical trial, only 1 of 20 patients had a partial response and significant irAEs were reported, including grade 3 neutropenia, grade 3 fatigue, nausea, diarrhea, headaches, vomiting, and weight loss [160]. Another orally administered TLR7 agonist called bropirimine was compared with bacillus Calmette-Guérin (BCG) intravesical immunotherapy (standard treatment for carcinoma in situ bladder cancer) in a pivotal phase III trial and showed lower levels of toxicity and treatment discontinuation (4% of patients in the bropirimine group withdrew vs. 14% in the BCG group). Unfortunately, antitumor efficacy was not sufficient to merit FDA approval [161].

Roche is currently developing orally available synthetic TLR7/8 agonists for advanced cancers. For example, the compound RO-7119929 is being tested in a phase I clinical trial in patients with metastatic hepatocellular carcinoma with the IL-6 inhibitor tocilizumab, the latter of which is to be administered in case of severe steroid refractory cytokine release syndrome. Additionally, Primmune Therapeutics is continuing where Anadys left off with two orally available TLR7/8 agonist prodrugs, PRTX-007 and PRX-034. Preclinical data show increased levels of type I IFNs, IL-6, and IL-1Ra upon incubating the prodrugs with human PBMCs, with no increases in IFN-γ, IL-2, or IL-12p70; these results indicate minimal engagement of the NF-κB proinflammatory pathway, which might minimize systemic toxicities [162].

Given the history of failed attempts at systemic delivery of synthetic TRL7/8 agonists, considerable effort has also focused on localized administration of these compounds to circumvent systemic toxicity [163]. Early work on local delivery evaluated a liquid formulation of R837, based on excipients such as poloxamers and β-cyclodextrin, for the treatment of non-muscle invasive bladder cancer [164]. A formulation called TMX-101 or Vesimune progressed through phase I and II trials, with promising results in patients with carcinoma *in situ* bladder cancer [165,166]. Among compounds that are currently in trials, VTX-2337, a benzazepine-based TLR8 agonist, was shown to be well tolerated in phase I and is currently in phase II trials in combination with Doxil (doxorubicin) for ovarian cancer treatment and with cetuximab for squamous cell carcinoma of the head and neck [167-169]. Intratumoral administration of MEDI-9197 (or 3M-052) was evaluated in a phase I clinical trial in combination with durvalumab, another anti-PD-1 antibody. Although the preclinical data were very promising, further development of this compound has been discontinued by AstraZeneca [170]. NKTR-262 is another TLR7/8 agonist being evaluated in the context of intratumoral administration in advanced cancers in combination with the CD122 agonist NKTR-214. Similarly, LHC165, a benzonaphthyridine TLR7 agonist adsorbed to aluminum hydroxide, is being tested in a phase I clinical trial with the anti-PD-1 antibody spartalizumab. Preclinical compounds that have shown promise in mitigating systemic toxicity by localized retention include ALT-702, TransCon-R848, and VX-001; ALT-702 is based on a depot-forming peptide technology and the latter two are described as “sustained-release R848 formulations.” These systems are discussed in greater detail in Section 4, where we focus on formulation and carrier-based approaches.

### 3.3 Systemic delivery of IMDs as Antivirals for Infectious Diseases

In addition to treatment of skin conditions as discussed in section 3.1, IMDs are being explored as systemic treatments for chronic infectious diseases such as hepatitis C (HCV), hepatitis B (HBV), and HIV. The development of orally available prodrug forms of TLR7/8 agonists has been extensively evaluated in the treatment of chronic infectious disease, with the goal of improving patient compliance and minimizing side effects [171]. As in the application to cancer, however, the transition from treating topical infectious conditions to chronic systemic infections has also been limited by narrow therapeutic indexes of systemically/orally administered TLR7/8 agonists. Low doses have insufficient therapeutic effects and higher doses are associated with vomiting, fatigue, lymphopenia, and fever, in addition to hepatic and renal impairment [172]. Although toxicity remains a concern, the antiviral state generated by type I IFN induction upon TLR7 ligation makes these agonists promising antiviral agents for chronic infectious diseases [173]. For example, type I interferons elicited by TLR7/8 activation can induce viral RNA transcript degradation and activation of protein kinase R, which blocks translation of viral mRNAs [174].

The first clinical attempts at treating chronic infectious diseases with synthetic TLR7/8 agonists focused on chronic HCV. Oral administration of R848 was extensively evaluated but failed in two phase IIa clinical trials because of toxicity at higher doses, similar to issues with PEGylated IFN-α injections, the standard treatment for chronic HCV [175]. Oral administration of PF-4878691 (852-A) was tested in a phase I trial in patients with chronic HCV but showed low bioavailability and highly variable absorption compared with parenteral administration. This drug was therefore discontinued for treatment of HCV but further evaluated in the context of advanced cancers [176]. ANA975 and ANA971 are oral prodrug formulations that are converted to active compound by first-pass metabolism [177]. They were also tested in phase I clinical trials in patients with chronic HCV but failed due to inadequate therapeutic indexes.

Although synthetic TLR7/8 agonists are no longer, to our knowledge, being tested for chronic HCV, treatments for chronic HBV have shown promising results. HBV infects hepatocytes in the liver without activating hepatic non-parenchymal cells such as Küpffer cells and liver sinusoidal endothelial cells or surrounding immune cells such as myeloid DCs (mDCs), pDCs, and macrophages [178]. TLR7/8 agonists play a role in preventing viral transmission and replication by mechanisms similar to those discussed earlier, including upregulation of IFN-stimulated genes (ISGs) and the generation of an antiviral state by type I IFN signaling [179]. One of the many lead compounds in the clinic, RO-7020531, has completed a phase I trial and is currently in phase II trials in combination with direct-acting antivirals [180-182]. This compound is a double prodrug of a TLR7 agonist that has shown promising preclinical results. In an AAV-HBV mouse model, RO-7020531 induced both innate pharmacodynamic responses and adaptive immune responses [183]. GS-9620 is another oral TLR7 agonist that was tested clinically for treatment of chronic hepatitis B. This compound exhibited high first-pass hepatic clearance and increased levels of type 1 IFN upon oral administration compared with intravenous administration, with similar systemic exposure in nonhuman primates (NHPs) [184]. At low doses, GS-9620 was shown to activate ISGs without inducing systemic interferons, which suggested the presence of a therapeutic window for inducing an anti-HBV response [185]. Ferrari and colleagues demonstrated sustained antiviral effects against woodchuck hepatitis virus (WHV) in a woodchuck model of HBV and against HBV in a chimpanzee model, with significantly increased production of IFN from monocytes rather than direct activation of antiviral pathways in hepatocytes. These findings indicated that GS-9620 could be used in concert with other standard antiviral treatments [186,187]. The same researchers observed significantly increased levels of antigen presentation in hepatocytes, which led to improved HBV-specific immune responses. In a phase 1b trial with 1 or 2 doses of oral GS-9620 administered 7 days apart, no change in serum HbsAg or HBV DNA levels were detected, despite induction of interferon-stimulated genes [185]. Clinical development of GS-9620 for chronic HBV has since been discontinued, but is still being examined for treatment of HIV (*vide infra*). APR002, a liver-targeted TLR7 agonist designed to act locally in the liver and gastrointestinal tract, is being developed by Apros Therapeutics. Preclinical studies in NHPs showed that retention in the liver is partly mediated by organic anion transporting polypeptide transporters [188]. These studies demonstrated sustained suppression of serum WHV DNA, lowered levels of serum WHsAg, and durable antibody responses in a woodchuck model of chronic WHV.

Bertoletti and colleagues probed purified hepatic immune cells from healthy and HBV-infected human livers with numerous TLR agonists and demonstrated that only TLR8 agonists were able to selectively activate liver-derived cells, resulting in substantial production of IFN-γ, which has been shown to mediate clearance of HBV in infected chimpanzees [189,190]. GS-9688, a selective TLR8 agonist currently in phase II clinical trials for treatment of chronic HBV, can activate TLR8-expressing gut and hepatic immune cells, leading to secretion of immune mediators in the portal vein [191]. An effective dose of 3 mg/kg was defined in WHV-infected woodchucks and 3 mg in virally suppressed and viremic patients with chronic HBV [192].

Beyond treatment of HCV and HBV, recent work has shown the potential of TLR7/8 agonists in reversing HIV latency (a state where cells are infected and contain an integrated and functional HIV viral genome, but where active viral transcription is silent). GS-9620, discussed earlier in the treatment of chronic HBV, is currently in a phase I trial for reversing HIV latency. Preclinical data indicated that TLR7/8 agonist-induced increases of IFN-α effectively inhibited HIV-1 replication *in vitro* in activated lymphocytes and macrophages [193]. Work done by Barouch and colleagues combining Ad26/MVA vaccination with oral TLR7 agonists GS-9620 or GS-986 (a GS-9620 analog) in an NHP model showed significantly reduced viral DNA in lymph nodes and peripheral blood [194]. They also observed improved virologic control and delayed viral rebound following discontinuation of antiretroviral therapy [195]. Whitney and colleagues demonstrated the ability of these oral TLR7 agonists to induce transient viremia in rhesus macaques (RMs) [196]. Thus, TLR7/8 agonists may have a role to play in HIV cure strategies.

Although we have focused here on viral infections, it is important to note the role that synthetic TLR7/8 agonists play as adjuvants in vaccines against bacterial infections. The mechanisms underlying TLR7/8 sensing of bacterial RNA are gradually being established [197-199]. Preclinical studies of these agonists in vaccine formulations against bacterial pathogens demonstrate the ability of these compounds to activate both neonatal and adult DCs alike thus also highlighting their potential in boosting neonatal immunizations [200-202].

### 3.4 Anti-inflammatories and bronchodilators for Respiratory Ailments

In addition to their utility as potential anti-viral compounds, synthetic TLR7/8 agonists are being explored as agents to shift the polarization of immune response and as bronchodilators for treatment of allergic conditions such as asthma and rhinitis. Allergic reactions are characterized by highly Th2-polarized immune responses and the production of allergen-specific IgE antibodies. One approach to treatment of such disorders has been to therapeutically shift the polarization of the immune response from pathology-causing Th2 to a non-pathologic Th1 response. Preclinical studies using R848 as a model compound have demonstrated its ability to activate DCs, airway epithelial cells, and Th1 cells, leading to Th1 immune polarization [203]. In animal models of allergy, R848 treatment decreased airway hyperreactivity, airway remodeling, and activation of airway nerves, leading to increased nitric oxide production [204]. R848 was also able to reverse airway reactivity to allergen challenge, thus preventing airway smooth muscle proliferation and goblet cell hyperplasia [205]. Initial use of synthetic TLR7/8 agonists in allergic asthma was motivated by work done on administration of CpG oligodeoxynucleotides (CpG ODN; TLR9 agonists) to airways, which were shown to induce a redirection of the immune response polarization from Th2 to Th1. Inappropriate Th2 responses to harmless environmental antigens need to be counterbalanced by a strong Th1 cytokine response to reduce the downstream effects of the allergy [206]. Asthma therapies have focused on addressing airway inflammation and excessive bronchoconstriction [207]. Preclinical work using murine models of asthma has shown the potential of synthetic TLR7/8 agonists to significantly reduce ovalbumin (OVA)-induced airway hyperreactivity and eosinophilic inflammation [208]. TLR7/8 agonists inhibit IgE synthesis in favor of IgA synthesis in human B lymphocytes and can reverse airway hyperresponsiveness by IFN-γ-mediated effects. Intracellular signaling through NF-κB and the AP-1 family of transcription factors is implicated as the mediator of this response *in vitro*, whereas acute bronchodilation effects are shown to be mediated via production of nitric oxide upon TLR7 activation.

Synthetic TLR agonists have also been evaluated for the treatment of allergic asthma and rhinitis clinically. GSK-2245035 was investigated in clinical trials for the treatment of allergic asthma and allergic rhinitis [209]. This compound was designed to preferentially upregulate IFN-α secretion without significant changes in TNF-α as a means to alter the immune microenvironment of the airway and thus to modify the Th2 response to aero-allergens [210]. Preclinical work on the development of the 8-oxo-adenine antedrug series, possessing an ester moiety that cleaves to an inactive acid in the presence of plasma esterases, allowed for lowered systemic toxicities [211]. AZD-8848 was the result of this selective antedrug TLR7 agonist library optimized for airway treatment [212]. However, AstraZeneca has since discontinued development of TLR7 agonists. VTX-1463, a selective TLR8 agonist, is currently undergoing phase I trials for treatment of allergic rhinitis. Preclinical data have shown significant upregulation of Th1 inflammatory mediators, such as IL-12, IFN-γ, and MCP-1, that shift the balance of the Th1:Th2 ratio toward lowering allergic effects [213].This compound was well tolerated in a phase I clinical trial for grass pollen allergy, with improved levels of total nasal symptom scores, the primary clinical endpoint [214].

## 4. Bioconjugation and other delivery strategies

The discussion above demonstrates the breadth of preclinical and clinical applications of synthetic TLR7/8 agonists, but also highlights the myriad unsuccessful attempts at clinical translation. Overall, two main problems have dominated failures in clinical trials: insufficient therapeutic efficacy and unacceptable toxicity (i.e., narrow therapeutic index). Therefore, significant research has focused on bioconjugation and other delivery strategies to overcome these limitations and realize the full potential of these synthetic small-molecule immunomodulators.

### 4.1 TLR7/8a conjugates to enhance vaccine efficacy

#### 4.1.1. Antigen-TLR7/8 conjugates

Some of the early research on improving the potency of vaccine formulations involved conjugating synthetic TLR7/8 agonists to vaccine antigens as a means to prevent the former from entering the bloodstream and to focus their effects precisely on antigen-specific B cells and those DCs that acquire antigen and are most relevant for T cell activation. The hypothesis was that covalent conjugation would allow for colocalization of antigen and adjuvant, thus greatly increasing the likelihood that the antigen and adjuvant are taken up by the same APC, which in turn could enhance antigen presentation to CD4^+^ and CD8^+^ T cells [215].

Work done by Seder and colleagues comparing the efficacy of R848 to that of the TLR9 agonist CpG ODN as vaccine adjuvants showed that R848 induced lower levels of antigen-specific CD4^+^ and CD8^+^ T cells and reduced IgG_1_ and IgG_2a_ responses to the HIV-1 Gag protein [216]. Using a reactive derivative of R848, the TLR agonist was conjugated to HIV-1 Gag protein, and the IMD-conjugated protein elicited elevated cytokine production by antigen-specific CD4^+^ and CD8^+^ T cells to the antigen. This study indicated that the discrepancy between innate activation of DCs and lack of T cell response from free R848 could be a delivery problem, wherein the timing of antigen presentation was not optimized with the activating effects of R848. Follow-up studies in rhesus macaques (RMs) comparing the TLR7/8 agonist-Gag protein conjugate with the Gag protein itself or with CpG ODN showed increased Th1 responses and Gag-specific CD8^+^ T cell responses for the conjugate [217]. The mechanism of action of the TLR7/8 bioconjugate was hypothesized to be due to enhanced migration of 6 distinct DC populations to dLNs, thus significantly increasing the total number of specific DC subsets in the dLNs. Increased uptake of OVA, the model antigen, was shown in the conjugate system compared with a mixture of unconjugated OVA and TLR7/8 agonist (37.8% vs. 8.8%, respectively), validating the above hypothesis [218]. This work supported that of Kedl et al., who showed that TLR7/8-antigen bioconjugates increased the sensitivity of responding T cells to low amounts of antigen [219].

An important issue in preparing antigen-IMD conjugates is ensuring that the IMD is not linked to the antigen at an important site for neutralizing antibody recognition. To this end, Hedestam and colleagues showed that attaching a TLR7/8 agonist onto surface-exposed lysine residues on the external subunit of the HIV-1 envelope glycoprotein gp120 led to reduced antibody recognition of the CD4 binding site, depending on the concentration of TLR7/8 agonist conjugated to gp120 [220]. Work by O’Hagan and colleagues also highlighted the potential loss of conformation that can occur with bioconjugation reactions in this context and noted that a disperse mixture of conjugated species can form due to the high number of equivalent surface-exposed amino acids available for conjugation [221]. These authors used an *ortho*-activated benzaldehyde instead of a non-substituted derivative to overcome the need to increase the reactivity of lysine amino groups, thus reducing the complexity of the reaction and purification steps and increasing the product homogeneity.

**Figure 4.**
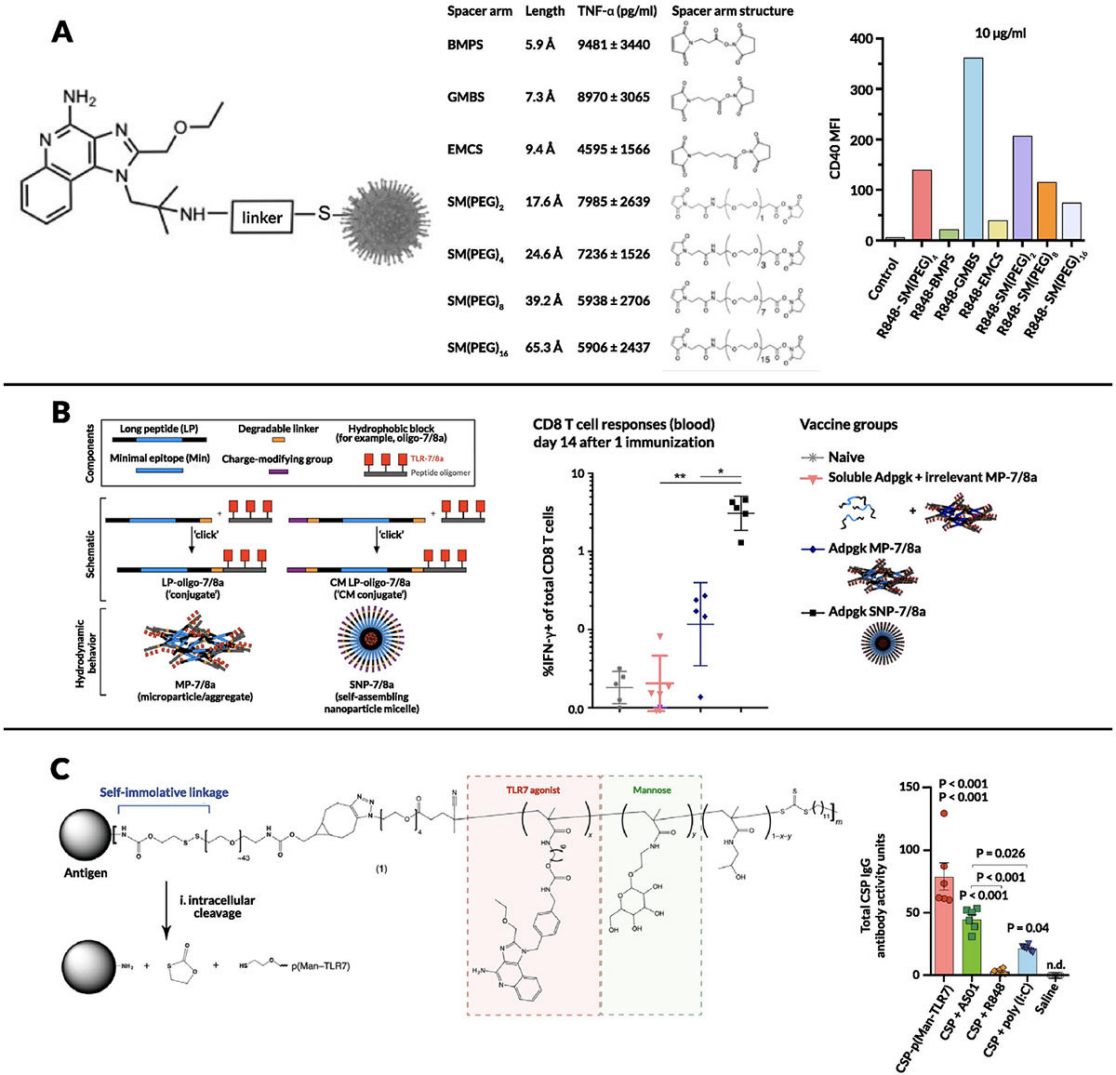
Bioconjugation approaches to enhance vaccine potency. (A) Dependence of R848-influenza conjugate particles on choice of linker strategy. (B) Charge-modified peptide strategy that results in particulate bioconjugates for personalized cancer vaccines. (C) Self-immolative linker approach for enhancing potency of malaria antigen-TLR7 conjugate.

The above bioconjugate studies all focused on linkage of IMDs to single protein antigens. Work done by Alexander-Miller and colleagues allowed for multiple antigenic targets (i.e., incorporating all viral proteins) by conjugating an amine-modified R848 onto reduced inactivated influenza virus A particles (IPR8) using an amine to sulfhydryl crosslinker featuring N-hydroxysuccinimidyl ester and maleimide groups that connect the amine of the drug and the thiols on the virus particles respectively (SM(PEG)_4_; Fig. 4A) [222]. This system was tested in the African Green Monkey (AGM) model of neonatal vaccination and resulted in a 10-fold increase in anti-influenza virus IgG levels, increased levels of influenza-specific IgM and IFN-γ^+^ influenza-specific T cells, and decreased viral load in the trachea. This improved response was again tied to an increase in the total number of DCs in the dLNs and increased DC expression of maturation markers such as CD80 and CD86 [223]. The R848-IPR8 conjugate was further tested for long-term responses in the AGM model and showed significantly increased levels of long-lived IgG antibody response 6 months after dosing [224]. Recent work from the same group explored the development of second-generation R848-IPR8 conjugates prepared via a two-step synthesis process [225]. First, R848 was modified with different crosslinker moieties to introduce maleimide groups that were then conjugated to the reduced virus containing thiol groups. These systems were tested in RAW264.7 cells for TNF-α production with the *N*-γ-maleimidobutyl-oxysuccinimide ester (GMBS, ThermoFisher) linker showing higher CD40 expression (by an order of magnitude) compared with both the unconjugated control and the first-generation SM(PEG)_4_ linker (Fig. 4A). These conjugate vaccine strategies highlight the necessity for improved formulation approaches in order to maximize the potential of TLR7/8 adjuvantation.

#### 4.1.2. Polymer and particulate formulations of TLR7/8 agonists

While the above examples demonstrate bioconjugation of TLR7/8 agonists onto soluble proteins, another approach pioneered by Seder and colleagues involves the intentional design of conjugate formulations that self-aggregate into larger particulates [226,227]. They attached a number of antigens or neoantigens using a charge modification conjugation approach that comprised peptide antigens linked to both a charge-modifying polypeptide and a hydrophobic polypeptide block through enzyme-degradable linkers at the N and C termini, respectively. Then, the oligopeptide-based hydrophobic blocks were linked to a precise number of TLR7/8 agonists. This work further strengthens the hypothesis that antigen form (soluble vs. particulate) is a critical aspect of CD8^+^ T cell immunogenicity. Vaccination with particle antigens, either covalently attached or mixed with adjuvant, led to 20-fold-higher CD8 T cell responses compared to soluble antigen, with the particle conjugates being retained longer in dLNs and showing higher uptake by CD11c^+^ DCs (Fig. 4B). Another example of this approach was demonstrated by Hubbell and colleagues using a reversible linker strategy [228]. In this system, protein antigens were conjugated via a cleavable linkage to a statistical copolymer containing mannose-binding receptors and a TLR7 agonist called P(Man-TLR7). Antigens were modified with an amine-reactive heterobifunctional bicyclononyne-decorated linker, which is sensitive to disulfide reduction (stable in serum and cleaves on endocytosis). Vaccination with a P(Man-TLR7) conjugate linked to CSP (circumsporozoite protein from *Plasmodium falciparum* malaria) induced a significant increase in CSP-specific CD8^+^ T cell response (TNF-α^+^ and IFN-γ^+^ T cells) compared with controls, including the adjuvant currently used in the malaria vaccine being studied in clinical trials (Fig. 4C).

**Figure 5.**
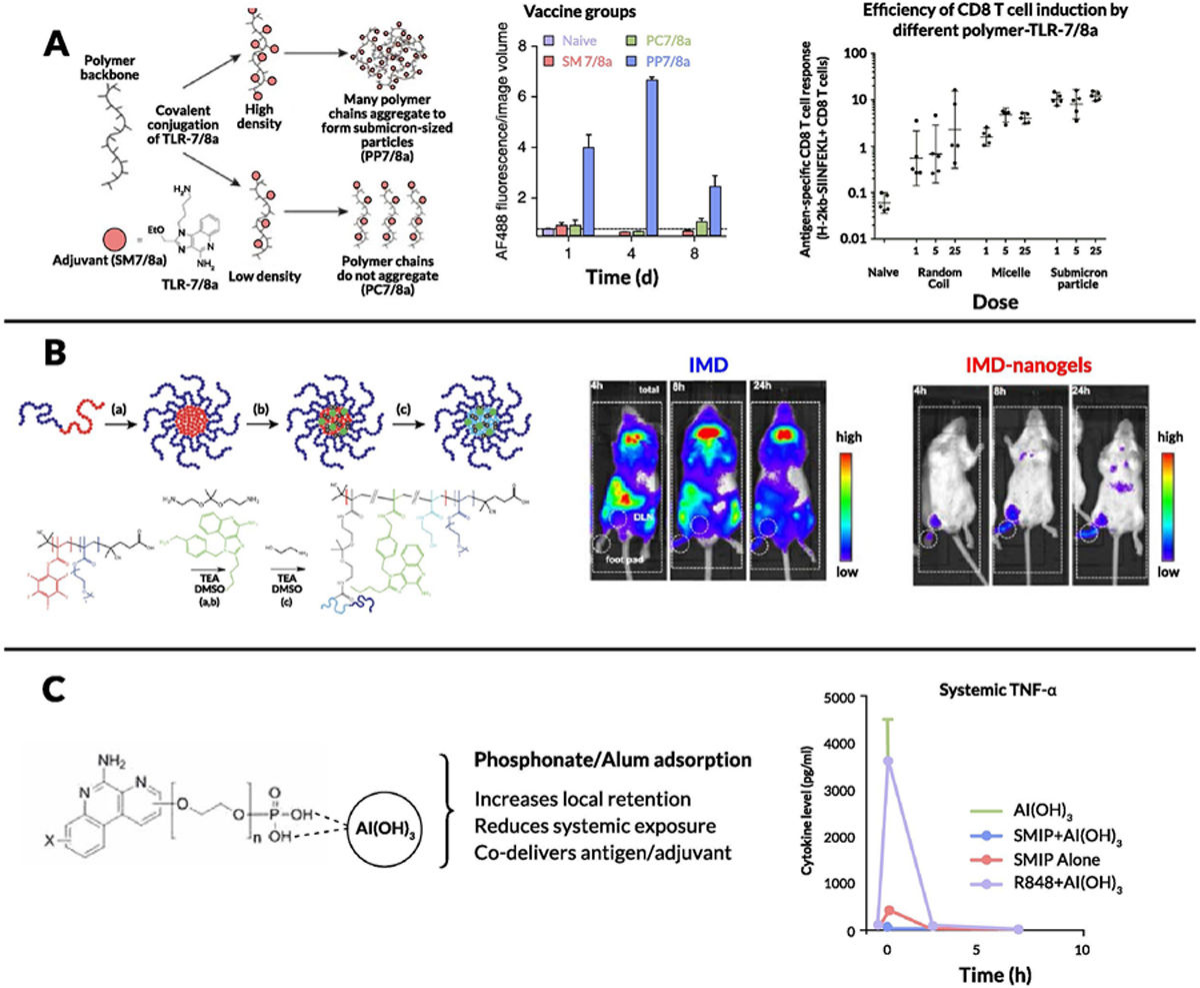
Polymer and particulate approaches to improve adjuvant potency. (A) Effect of varying TLR7 agonist density on lymph node residence time of the particle and varying size and architecture of the polymer on antigen-specific T cell response. (B) polymeric nanogel approach toward enhanced lymph node delivery and retention. (C) Adsorption to alum resulting in ‘depot’ effect which minimizes conc of TNF-α in the blood upon administration.

The rationale for particulate formulations has been bolstered by extensive work by Seder and colleagues, wherein TLR7/8 agonists were covalently conjugated onto synthetic polymer scaffolds to further explore the mechanistic effects of different linker structures and TLR7/8 agonist densities on the physicochemical properties of the prodrug particles [229]. Particulate TLR7/8 conjugates showed enhanced lymph node cytokine production and uptake by migratory APCs as well as an order of magnitude increase in the influx of CD11c^+^ DCs and monocytes in the dLN compared with soluble forms (Fig. 5A). These authors probed further into how the carrier size and morphology of these conjugates correlate with immune activation by comparing 3 types of carriers: a random coil polymer (~4 nm), a micelle nanoparticle (~10 nm), and a sub-micrometer particle (~300 nm) [230]. A significantly increased CD8^+^ response for the sub-micrometer particles as well as direct correlations between hydrodynamic radii and magnitude of lymph node IL-12 production as well as uptake by APCs were observed (Fig. 5A). Interestingly, the uptake of the sub-micrometer particles was 5-fold higher for monocytes and macrophages than for DCs, suggesting that monocytes and/or macrophages play a crucial role in adjuvant activity in this size range. Particulate polymer or peptide formulations thus represent a promising approach to improve LN accumulation and retention of these agonists.

In a similar vein, De Geest and colleagues used bis-amino-ketal 2,2-bis(aminoethoxy)propane cross-linkers to create amphiphilic copolymer nanogels that were attached to IMD TLR7/8 agonists [231,232]. Compared to free TLR7/8a, immunization with TLR7/8a-conjugated nanogels led to increased internalization by multiple subsets of immune cells in LNs including DCs, B cells, macrophages, and monocytes, with the vast majority of the nanogel localized to the subcapsular and medullary sinuses of the lymph node (Fig. 5B). The same group developed lipid-polymer amphiphiles consisting of cholesterol as a lipid motif and a hydrophilic polymer conjugated to a TLR7/8 agonist [233]. They demonstrated that this polymer binds to albumin with high affinity (as assessed using bio-layer interferometry), allowing for highly efficient passive translocation to lymphoid tissue via “albumin hitchhiking” and leading to a significant increase in DCs in the dLN along with high expression of CD80 and CD86. This lymph node targeting approach was verified using IFN-β reporter mice, with bioluminescent imaging showing minimal systemic activation with a strong signal in the dLN. David and colleagues conjugated a TLR7/8 agonist onto hyaluronic acid via a 2-chloro-4,6-dimethoxy-1,3,5-triazine-activated amidation strategy, which resulted in an immunologically “silent” conjugate that was activated via proteolysis or enzymatic cleavage of the amide bond [234]. Upon co-administration of the conjugate with diphtheria toxoid (CRM197) as a model antigen, they observed a significant increase in antigen-specific IgG titers after a single boost. Kishimoto and colleagues showed that conjugating R848 onto polymer particles via an acid-labile bond resulted in an order of magnitude increase in antibody titer (Th1 focused; i.e., higher IgG_2c_/IgG_1_ ratio), increased antigen-specific T cells, and a significant influx of myeloid DCs, granulocytes, and macrophages into the dLN [235]. Another synthetic conjugate approach toward increasing DC activation and internalization in dLNs was shown by Hong and colleagues, wherein a TLR7/8 agonist with a terminal alkyne moiety was conjugated onto azide-coated iron oxide nanoparticles (NPs) through copper-catalyzed azide-alkyne cycloaddition and co-administered with OVA [236]. The resultant increased antigen-specific T cell activity reinforced the use of the synthetic conjugation approach as a method to improve vaccine potency. Likewise, Appel et al. conjugated a TLR7/8 agonist onto a copolymer of dimethyl acrylamide and neopentyl glycol diacrylate formulated with beta-cyclodextrin [237]. Upon co-injection with either OVA or gp120, the adjuvant formulation exhibited significantly improved IgG_2c_ antibody titers.

An alternate approach to promote lymph node accumulation and reduce systemic dissemination of TLR7/8a adjuvants is to engineer their binding to existing clinical adjuvants such as alum. A team at Novartis pursued this approach by screening TLR7 agonists functionalized with polyethylene glycol (PEG) linkers and terminal phosphonate groups [238]. The PEG linkers allowed for increased solubility at neutral pH while the anionic phosphonate functional groups facilitated efficient adsorption to alum (Fig. 5C). Alum-bound TLR7 agonist adjuvants were tested in multiple vaccination mouse models; they demonstrated enhanced activation of APCs, priming of IFN-γ^+^CD4^+^ T cells, and, most importantly, antigen-specific B cell responses (both antibody-secreting and memory phenotypes) [239-243]. Alum-TLR7 conjugates were subsequently evaluated in a phase I clinical trial with the antigen present in the Menjugate (meningococcal group C–CRM197 conjugate) vaccine. In this study, the highest dose (100 μg) caused severe irAEs in one-fifth of patients, which correlated with high plasma levels of TLR7 agonist, while lower doses were deemed safe and effective in eliciting antibody responses similar to Menjugate (which contains alum as an adjuvant), though a larger-scale clinical trial was deemed necessary for verification [244]. TLR7 agonists have also been adsorbed to alum through mixing lipid tail-modified 3M-052 with phospholipids to generate very small lipid nanoparticles, which subsequently adsorb to alum via phosphate groups [245]. Upon co-injection of these alum/lipid nanoparticle-TLR7a complexes with a tuberculosis vaccine antigen, improved Th1 responses were observed relative to free 3M-052, with increased levels of antigen-specific CD4^+^ T cells and high levels of serum antibodies, which raises the question of whether there is potential synergy between TLR7/8 agonists and alum beyond simply a depot effect. This concept would be interesting to explore further, given that Seder and colleagues noted that adjuvant efficacy is lowered when TLR7 is co-formulated with an MF59-based nano-emulsion instead of alum, despite prior studies showing that MF59 induces higher binding titers than alum alone [246-248]. More recently, an Alum/TLR7a-adjuvanted whole-virion inactivated SARS-Cov-2 vaccine received emergency approval by India’s Central Drugs Standard Control Organization at the end of 2020 [249-254]. At the time of writing this review, this vaccine has been administered to over ten million people, further underscoring the importance of formulated TLR7/8 agonists as vaccine adjuvants.

In addition to alum adsorption, a commonly studied approach for formulating IMDs has been through their encapsulation with or without vaccine antigens in biodegradable poly(lactic-co-glycolic acid) (PLGA) nanoparticles or other polymeric NPs. Pulendran and colleagues encapsulated soluble gp140 Env and lipophilic TLR7/8 agonist 3M-052 in PLGA NPs and showed an adjuvant effect comparable to that of alum-TLR7 and significantly higher than that of alum alone in RMs [255,256]. They demonstrated increased levels of Env-specific IgG antibodies in serum and vaginal secretions, high levels of neutralizing antibodies, and upregulated expression of CD86 on monocyte subsets 2 weeks after vaccination. Another interesting finding was the enhanced protection observed in young and adolescent RMs compared with older RMs, which could tie in to work done by Levy and colleagues that showed potent activity of TLR7/8 agonists on neonatal macaque blood cells [257]. TLR7/8 PLGA-based vaccine formulations have also been studied in the context of mucosal and tumor immunizations [258,259]. Other polymeric systems include block copolymers, developed by Wilson and colleagues, decorated with pyridyl disulfide ethyl methacrylate moieties for conjugation of thiol-containing antigen and a fatty acid-mimetic core for encapsulation of TLR7 agonist [260]. Vaccination with these block copolymer nanoparticles carrying antigen (OVA) and TLR7 agonist elicited increased levels of antigen-specific CD8^+^ T cells in bronchoalveolar lavage fluid, lung vasculature, and lung interstitium in addition to enhanced antigen-specific IgG antibody titers upon intranasal administration compared to free protein/TLR7a immunization. In a similar approach, Levy and colleagues formulated a block copolymer system based on PEG-*block*-polypropylene-sulfide (PPS) polymersomes, encapsulating a TLR8 agonist that served as a potent adjuvant system when co-loaded with antigen [261]. Encapsulation in polymers has also been used to generate novel “needle-free” vaccination strategies, which represent potentially promising avenues for vaccine formulations based on TLR7 agonists [262-264].

### 4.2 Toward safe, systemic delivery via drug carrier approaches

As noted above, systemic toxicities associated with synthetic TLR7/8 agonists are a major roadblock to the clinical success of these compounds. Several drug carriers have been used in attempts to solve these issues, including PEGylation and other conjugation approaches, nano- and micro-particles, stimuli-responsive release, and molecular targeting moieties.

**Figure 6.**
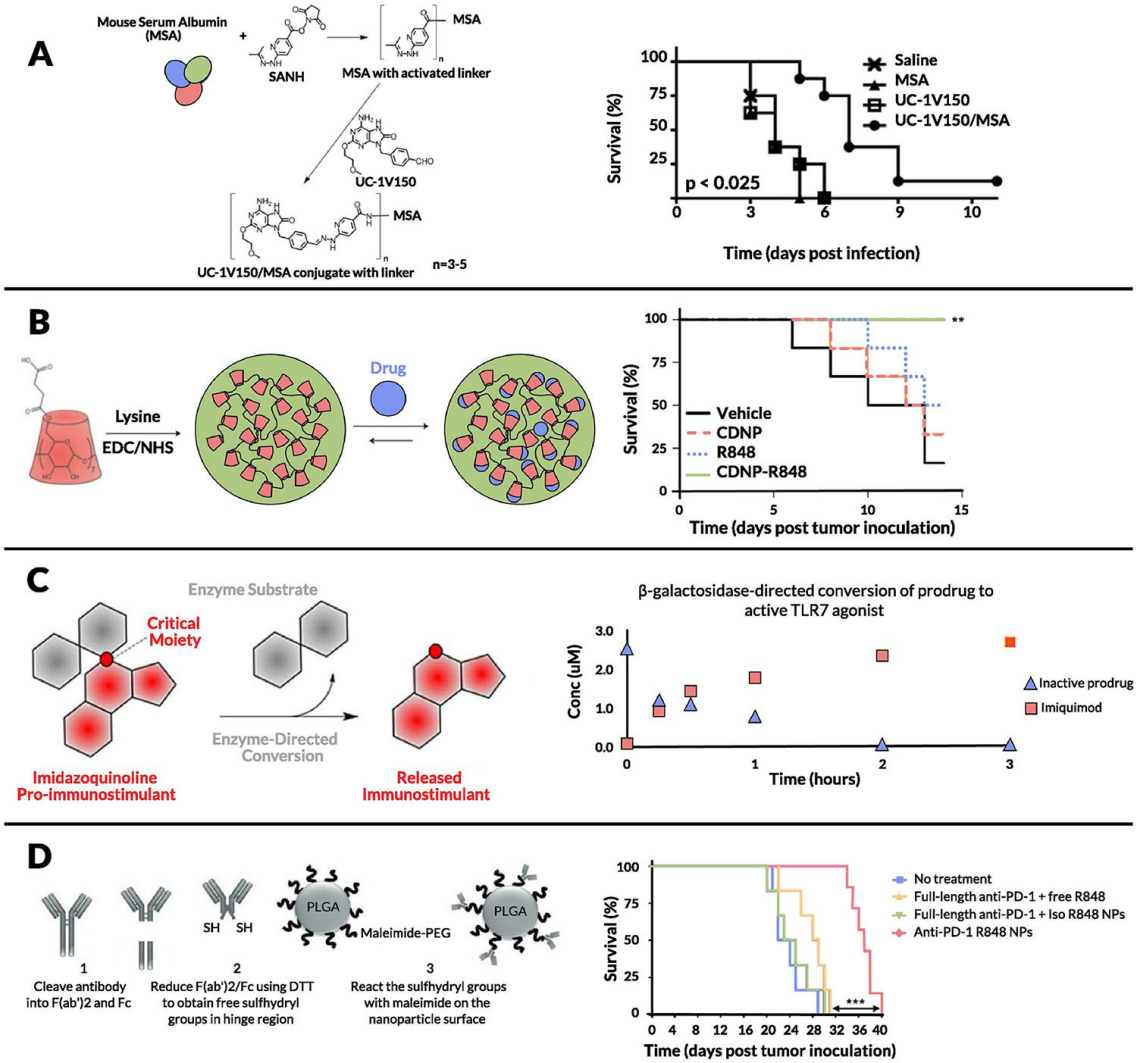
Toward systemic delivery of synthetic TLR7/8 agonists. (A) TLR7/8 conjugation onto carrier protein (mouse serum albumin) significantly improves survival in a pulmonary infectious disease model of *B anthracis*. (B) Cyclodextrin nanoparticle (CDNP) formulation of R848 significantly improves survival in the MC38 colon cancer mouse model. (C) β-galactosidase enzyme-mediated tuned release of imiquimod (R837). (D) PD-1 targeting approach localizes effect of R848 NPs upon i.v. administration and significantly improves survival in mice bearing MC38 tumors.

Carson and colleagues made a surprising observation when investigating PEGylation of TLR7 agonists. Specifically, while PEGylation improved solubility of these compounds, cytokine production *in vitro* was significantly lowered [265]. This loss of activity upon PEGylation was intriguing and the influences of PEG chain length (from 6 to 470 repeat units) on *in vitro* and *in vivo* effects of TLR7 ligands were examined. Short-chain PEGs displayed low potency to stimulate bone marrow-derived macrophages; however, when chain length exceeded 47, potency was restored, suggesting that spatially constrained conjugate ligands are limited in their ability to activate TLR7. Furthermore, conjugates with an amine end group were more potent than those ending with carboxyl. These data showing that conjugates using longer PEGs have improved solubility, circulation time, and plasma concentration, as well as increased IL-6 and TNF-α production *in vivo*, tie in nicely with previous observations that innate immune activity of PEG-TLR7 conjugates is affected by steric hindrance of the PEG linker [266]. The same group has shown that upon conjugation of a TLR7/8 agonist onto a carrier protein (mouse serum albumin) via a succinimidyl 6-hydrazone nicotinamide acetone hydrazone linker, the immunostimulatory potential of the agonist was significantly enhanced (Fig. 6A) [267]. Further work on TLR7 conjugation onto primary amine-functionalized Ficoll (400 kDa) or dextran (70, 500, and 2,000 kDa) using benzoic acid functional groups showed higher potency (by an order of magnitude) of TNF-α and IL-6 induction *in vitro* [268]. It is interesting to note that 12 nm dextran conjugates with similar conjugation ratios showed 10-fold higher potency than 37 nm dextran conjugates, whereas 29 nm linear dextran showed 10-fold higher potency than 14 nm spherical Ficoll conjugates. These observations were validated *in vivo* along with admixed controls, wherein the synthetic conjugates were at least 500-fold more potent at inducing production of cytokines such as IL-6, TNF-α, and IFN-γ than the unconjugated TLR7 ligand. These studies reinforce the idea that size, composition, and molecular architecture of synthetic TLR7 conjugates significantly affect immune potencies due to efficiency of uptake by APCs which is required for the generation of an adaptive immune response. These studies culminated in the development of 1V270, a phospholipid-conjugated TLR7 agonist that spontaneously self-assembles into 110–120 nm liposomes [269]. This liposomal formulation was tested in multiple murine tumor models, where NK cells were responsible for early efficacy and CD8^+^ T cells were critical for sustained inhibition of lung metastasis.

Beyond molecular bioconjugates, numerous nano- and microparticle-based encapsulation approaches have been developed to enable intravenous delivery of TLR7 agonists. Ainslie and colleagues encapsulated R848 in acetalated dextran microparticles and showed efficacy in treating visceral leishmaniasis, a systemic parasitic disease [270]. Another significant stride toward systemic administration of TLR7/8 agonists was made by Weissleder and colleagues [271-273]. They developed a single-cell high-content screening approach to identify compounds that could alter the polarization of macrophages from an M2 (wound-healing phenotype) to M1 (inflammatory) state *in vitro* [271]. Among the compounds tested, R848 was the most potent driver of macrophage re-education and they subsequently designed cyclodextrin NPs to encapsulate R848 based via host–guest interactions. Cyclodextrin NPs carrying R848 exhibited efficacy in both the MC38 colon cancer and B16.F10 melanoma models upon intravenous administration (Fig. 6B). Notably, toxicity was not addressed in this initial study; in a follow-up study, the same group attached adamantane to R848 via the tertiary alcohol group thus increasing the host-guest binding affinity and improving the stability of the drug-loaded supramolecular complex. They obtained antitumor efficacy with lower weight loss than with R848-cyclodextrin NPs [272]. Another recent attempt at intravenous administration of TLR7/8 agonists was reported by Kabanov and coworkers; they encapsulated R848 in poly(2-oxazoline) particles and showed improved survival rates with low toxicity in a metastatic, orthotopic lung adenocarcinoma model [274]. They attributed the antitumor effects to higher levels of Ly6C^+^ monocytes and CD8^+^ T cells in the tumor microenvironment. Moving beyond polymeric systems, Bourquin and colleagues demonstrated that gold NPs coated with a mixture of 1-octanethiol and 11-mercaptoundecanesulfonic acid encapsulating R848 via hydrophobic interactions was a potent delivery vehicle, showing improved lymphatic accumulation and antitumor effects in the CT26 colon cancer mouse model [275].

Stimuli-responsive nanomaterials are ubiquitous in the field of drug delivery and attempts to use this paradigm to minimize toxicity of TLR7/8 agonists have yielded fruitful results in preclinical studies. Hubbell and colleagues formulated TLR7 agonists in oxidation-sensitive polymersomes based on PEG-b-PPS, which would specifically release TLR7a on exposure to the oxidative environment within antigen presenting cell endosomes [276]. They observed enhanced DC uptake and activation and proinflammatory cytokine secretion by these polymersomes compared to free TLR7a cultured with DCs *in vitro*. They hypothesized that this enhanced response was due to NOX-2-dependent reactive oxygen species in DC early endosomes and lysosomes triggering burst release of the encapsulated TLR7 agonist. Other oxidation-sensitive systems have been reported, including one developed by Broaders and colleagues that is based on a dextran polymer with stable boronic ester groups [277]. Light is another stimulus that has been extensively studied in the context of responsive biomaterial systems. Esser-Kahn and colleagues reported the use of 2-(2-nitrophenyl)-propyloxycarbonyl, a photocleavable protecting group, to form a carbamate linkage with the C4-amines in R837 and R848, demonstrating release of the free drugs in response to 360 nm light [278]. Mancini et al. attached a β-galactopyranoside to R837 covalently at the C-4 amine and reported β-galactosidase-mediated immune cell activation (Fig. 6C) [279]. This enzyme-responsive activity represents another promising step toward synthetic TLR7/8 agonist-based formulations that can preferentially release active cargo based on specific cues in the tumor microenvironment. The same group furthered this work by developing glycosidase-directed R848 prodrugs, which release free R848 based on cancer cell metabolism [280]. Another recent attempt at enzyme-responsive release was demonstrated by Shi and colleagues, wherein a TLR7/8 agonist was conjugated to PEG via benzyl carbamate residues capped by a β-glucuronidase (β-GUS)-sensitive glucuronide. The conjugates were then systematically studied by varying the number of benzyl repeat units (GL1, 2, or 3) and the molecular weight of PEG (0.75, 2, and 5 kDa) [281]. They found that PEG5k-GL2-IMD self-assembled into vesicular NPs and demonstrated native drug release in response to esterase and β-GUS.

Particulate formulations of TLR agonists favor uptake by APCs due to their natural propensity to phagocytose foreign material; however, enhancing delivery to these key cells and/or directing uptake by specific immune cell subsets is another strategy under investigation. One approach to enhance the concentration of IMDs in tumors is to target TLR7/8a-loaded nanoparticles to T cells circulating in the blood, which subsequently hone into tumors: Schmid et al. demonstrated this strategy by functionalizing TLR agonist-loaded PLGA NPs with anti-PD-1 antibody fragments (Fig. 6D) [282]. These fragments allow for preferential targeting of activated PD-1^+^ T cells in the circulation and tumors, leading to modestly enhanced antitumor efficacy of R848 in multiple mouse models. To more directly target DCs, Figdor and colleagues developed PLGA NPs coated with PEG-lipids displaying antibodies targeting the DC marker DC-SIGN [283]. They observed a significant increase in binding and uptake by human monocyte-derived DCs and increased expression of CD80 and TNF-α *in vitro*. DEC-205 is another marker of cDC1 dendritic cells; i.v. infusion of DEC-205-targeted NPs in mice elicited significantly lower levels of systemic type I IFNs compared with soluble TLR7 agonists, while still inducing potent cytotoxic T cell responses following OVA immunization. Edwards and colleagues conjugated a TLR7 agonist (UC-1V50) to an anti-hCD20 antibody, rituximab, through the use of a bifunctional *N*-hydroxysuccinimide (NHS) linker. They demonstrated a significant increase in IL-12p40 secretion from RAW264.7 macrophages and specific binding to CD20^+^CD19^+^ B cells *in vitro* [284]. Andresen and colleagues used maleimide functionalized liposomes attached to murine anti-human DCIR (dendritic cell immunoreceptor) monoclonal antibodies (IgG_1_ specific for a nonhuman epitope) via Traut’s reagent (i.e., 2-iminothiolane) [285]. They observed preferential uptake of the nanoparticles by monocytes and mDCs when evaluated *in vitro* in PBMCs and significantly enhanced secretion of inflammatory cytokines such as IL-6 and TNF-α. Beyond active targeting moieties, some promising attempts have been made to bias uptake toward specific immune cell subsets or tissues by modifying the physicochemical properties of the carrier. Jensen and colleagues tested a TLR7 agonist encapsulated in liposomes composed of neutral, negatively charged, or positively charged phospholipids and found that positively charged formulations effectively biased the NPs to CD14^+^ monocytes in human whole blood, demonstrating a higher level of IL-12p40 secretion in these monocyte subsets and differentiation into CD14/DC-SIGN^+^ DCs, which are potent APCs that can stimulate both CD4 and CD8 T cell responses [286]. Neither anionic nor neutral liposomes could produce these effects, demonstrating potential for charge-based cellular targeting, which has been highlighted recently in several other studies [287,288]. These immune-cell biasing approaches in tandem with the other strategies described above could play a key role in making systemic administration of synthetic TLR7/8 agonists a clinical reality.

### 4.3 Localized (in situ) delivery approaches to improve intratumoral efficacy

Although systemic administration remains the most common approach for delivery of anticancer therapies, significant advances in interventional radiology that allow for minimally invasive access to almost every organ in the body such that several malignant lesions can be treated simultaneously or sequentially have shifted clinical trends toward local administration [289,290]. Following the FDA approval in 2015 of intratumoral delivery of talimogene laherparepvec (T-VEC), an oncolytic viral therapy [291], key discoveries have been made in intratumoral delivery that have encouraged further preclinical and clinical development of this route of administration. Such local therapy is capable of eliciting systemic anti-tumor immunity, as tumor cell killing stimulated in the treated tumor leads to antigen presentation in tumor-draining lymph nodes, and priming of new T cell responses against the tumor, a concept now referred to as “*in situ* vaccination.” These tumor-specific T cells traffic to the treated tumor site as well as to distal, untreated tumors [292], enabling systemic tumor regression following a localized treatment [293,294].

**Figure 7.**
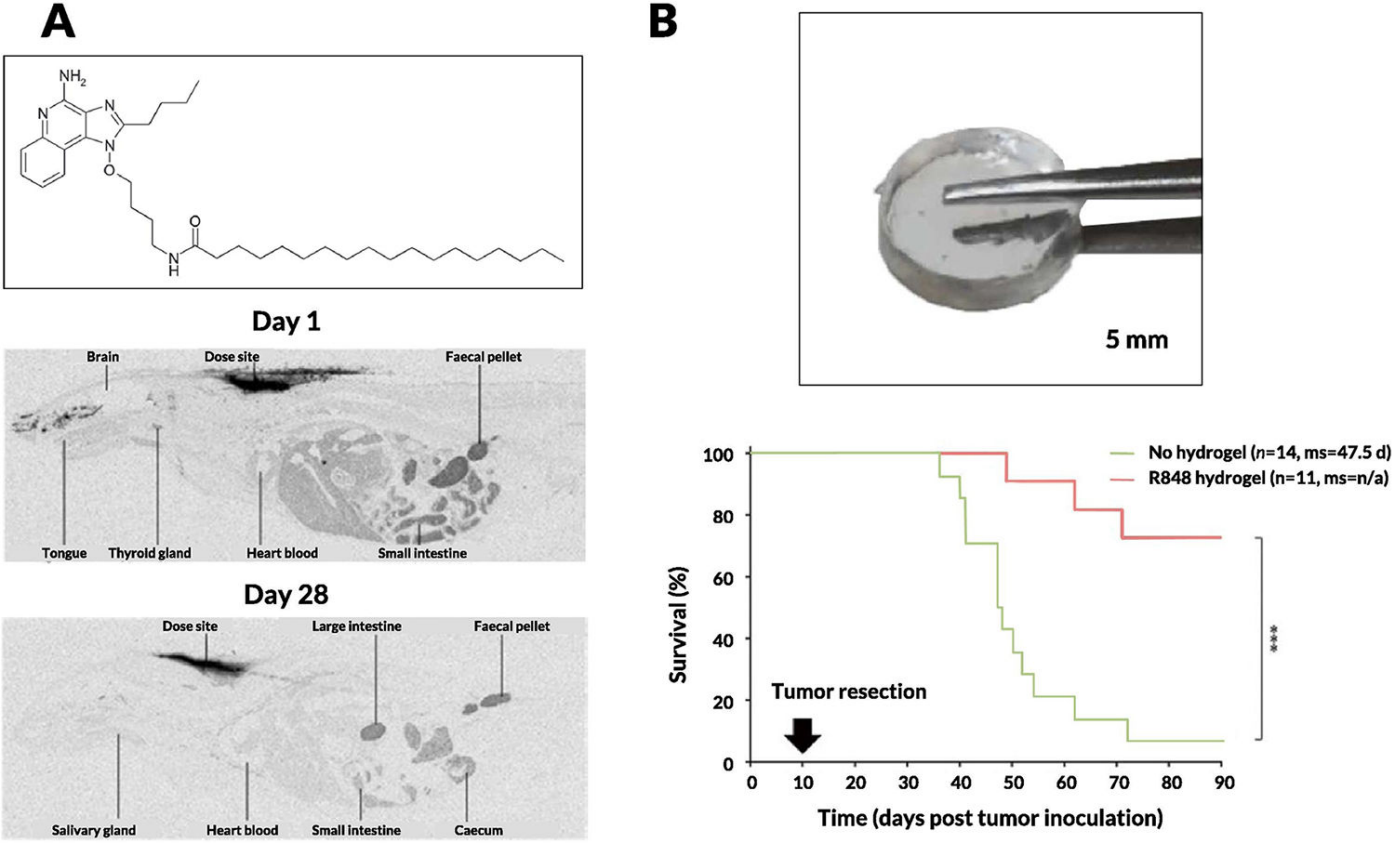
Localized approaches to maximize intra-tumoral efficacy. (A) C-18 lipid moiety of 3M-052 improves retention at injection site for up to 28 days. (B) HA-R848 scaffold, administered immediately post tumor resection, significantly improves survival in 4T1 mouse model of metastatic breast cancer.

In Section 3.2, we briefly discussed localized delivery approaches for TLR7/8 agonists that have already made their way to the clinic. In particular, 3M-052/MEDI-9197, an imidazoquinoline bearing a C18 lipid moiety, has been extensively evaluated preclinically for antitumor efficacy as a monotherapy and in combination with checkpoint blockade, OX-40 agonist antibodies, and other anticancer therapies (Fig. 7A). Despite the minimal systemic immune activation observed in mouse models, however, the phase I clinical trial showed systemic toxicities following intratumoral administration, with most patients experiencing pyrexia, fatigue, chills, decreased lymphocyte count, nausea, injection site pain, and cytokine release syndrome [295]. Thus, although the C18 lipid moiety did lower systemic toxicity, it was not enough to justify phase II trials. Schwarz and colleagues demonstrated that a Poloxamer 407 thermogel formulation of 3M-052 showed a significant improvement in tumor pharmacokinetics, with 20% of the drug still present 2 weeks after intratumoral injection, as well as decreased tumor burden and improved survival in mice bearing melanoma tumors [296]. An initial spike in serum drug concentration still occurred at 6 h post injection, however, indicating that further optimization was necessary to localize the drug in the tumor microenvironment. Wightman et al. showed an improved localized response when 3M-052 was formulated in liposomes, indicating potential for nano-formulations to localize the effect of synthetic TLR7/8 agonists in the tumor microenvironment [297]. Other localized TLR7/8 agonists discussed in Section 3.2 include NKTR-262, which is based on a 4-arm PEG star polymer to which 4 TLR7/8 agonists are attached via a hydrolysable glycine linker; this construct showed improved intratumoral retention and lowered plasma concentration compared to free drug. Alum-TLR7 was further optimized by Wu and colleagues via a medicinal chemistry approach to allow for phosphonate modification and maximum alum adsorption to minimize systemic dissemination [298]. One of these compounds, LHC165, is currently in phase I trials in combination with anti-PD-1 spartalizumab, administered intratumorally bound to alum [299]. In terms of preclinical formulations in industry, ALT-702 uses a depot-forming peptide with a fluorocarbon tail conjugated to a TLR7/8 agonist; it showed improved intratumoral retention and minimal systemic levels of inflammatory cytokines [300]. Finally, Ascendis Pharma is developing a long-acting prodrug of R848 formed by conjugation of R848 onto hydrogel microbeads via proprietary linkers [301].

Beyond these formulations advancing to the clinic via industry, some promising preclinical platforms have emerged from academic research that have also been able to localize the effect of TLR7/8 agonists using biomaterial-based strategies. De Geest and colleagues developed amphiphilic di-block copolymers that self-assembled into NPs [302] that were bound to a TLR7/8 agonist and cross-linked with pH-sensitive bis-amino-ketals via amide bond formation between reactive PFP esters and primary amines of the TLR7/8 agonist and crosslinker. Upon peritumoral administration, they observed lower tumor burden in the B16 tumor model at levels similar to that of free TLR7/8 agonist but without the systemic side effects. Lim and colleagues developed squalene emulsions encapsulating R848 in oleic acid [303]. Upon intratumoral administration, they observed lowered systemic IL-6 and significantly improved local IL-6, MCP-1, and MIP-1α kinetics. Forrest et al. formulated α-tocopherol-modified R848 with tocopherol-modified hyaluronic acid using an emulsification-solvent evaporation method [304]. Upon intratumoral administration in 6 canines with mast cell cancer, 4 demonstrated a reduction in tumor burden, and lesions disappeared in 1 animal. Recent work by Johnson et al utilized ring opening metathesis polymerization to synthesize injectable polynorbornene (pNb)-based triblock bottlebrush copolymer hydrogels that localize the effect of R848 therapy upon intra-tumoral administration to minimize systemic side effects [305]. Here, it was shown that co-delivery of R848 with paclitaxel in optimized multicompartment hydrogels led to enhanced cure rates and reduced toxicity compared to free drugs and nanoparticle formulations in mice bearing CT26 tumors. Nuhn and colleagues also utilized ring opening metathesis polymerization to synthesize (pNb-PEG)-(pNb-pentafluorophenyl) micelles which were covalently conjugated to a TLR7 agonist and cross-linked with pH-responsive ketal bisamines to obtain nanogels [306]. Interestingly, the nanogels showed dose-dependent activation of RAW 264.7 cells, whereas the soluble chains obtained upon degradation of the nanogel were immunologically silent; that is, they did not show TLR7 activity. Finally, Zhang and coworkers demonstrated that platelet-membrane coated NPs carrying R848 improved therapeutic efficacy in the MC38 colon cancer model upon intra-tumoral administration as a result of enhanced bioavailability in the tumor [307].

In addition to these particulate formulations of TLR agonists, another paradigm is to implant drug-releasing biomaterial matrices into a tumor resection site to achieve sustained and localized TLR signaling at the site of surgical resection. For example, Goldberg et al. developed a biodegradable hydrogel scaffold prepared from crosslinked hyaluronic acid (HA) that entrapped R848 [308]. In murine models of surgical implantation of TLR agonist-loaded gels into tumor resection sites, they observed no local tumor recurrence for at least one month and significantly improved survival, with the efficacy dependent on type I IFN signaling, NK cells, and CD4 and CD8 T cells (Fig. 7B). Interestingly, the authors showed that these antitumor effects were observed only upon local administration of the scaffold, with minimal survival following intravenous injections or a local bolus dose of the free drug.

### 4.4 In search of synergy: combination delivery approaches

The combination of synthetic TLR7/8 agonists with other therapies offers significant potential to further enhance their potency. Combination immunotherapies are particularly ubiquitous in the context of cancer, with countless preclinical investigations and more than 1800 ongoing clinical trials in the United States alone combining immune-checkpoint blockade with other immunotherapies. TLR7/8 agonists are showing promising preclinical results in overcoming tumor resistance to checkpoint blockade [309-311]. In the vaccine realm, work by Pulendran and coworkers demonstrated that the live attenuated yellow fever vaccine, one of the most effective immunological interventions worldwide, administered to over 400 million people, in fact activates multiple TLRs on DCs to elicit an immune response [312]. It has long been known that TLR agonists can exhibit synergistic activation of DCs; for example, R848 combined with either a TLR4 agonist or a TLR3 agonist allowed synergistic stimulation of inflammatory cytokines in multiple human DC subsets [313]. It is likely that TLR7/8 activation may synergize with multiple innate immune sensors or other immunostimulatory pathways beyond just TLRs to potentiate vaccine or immunotherapy responses [314,315].

**Figure 8.**
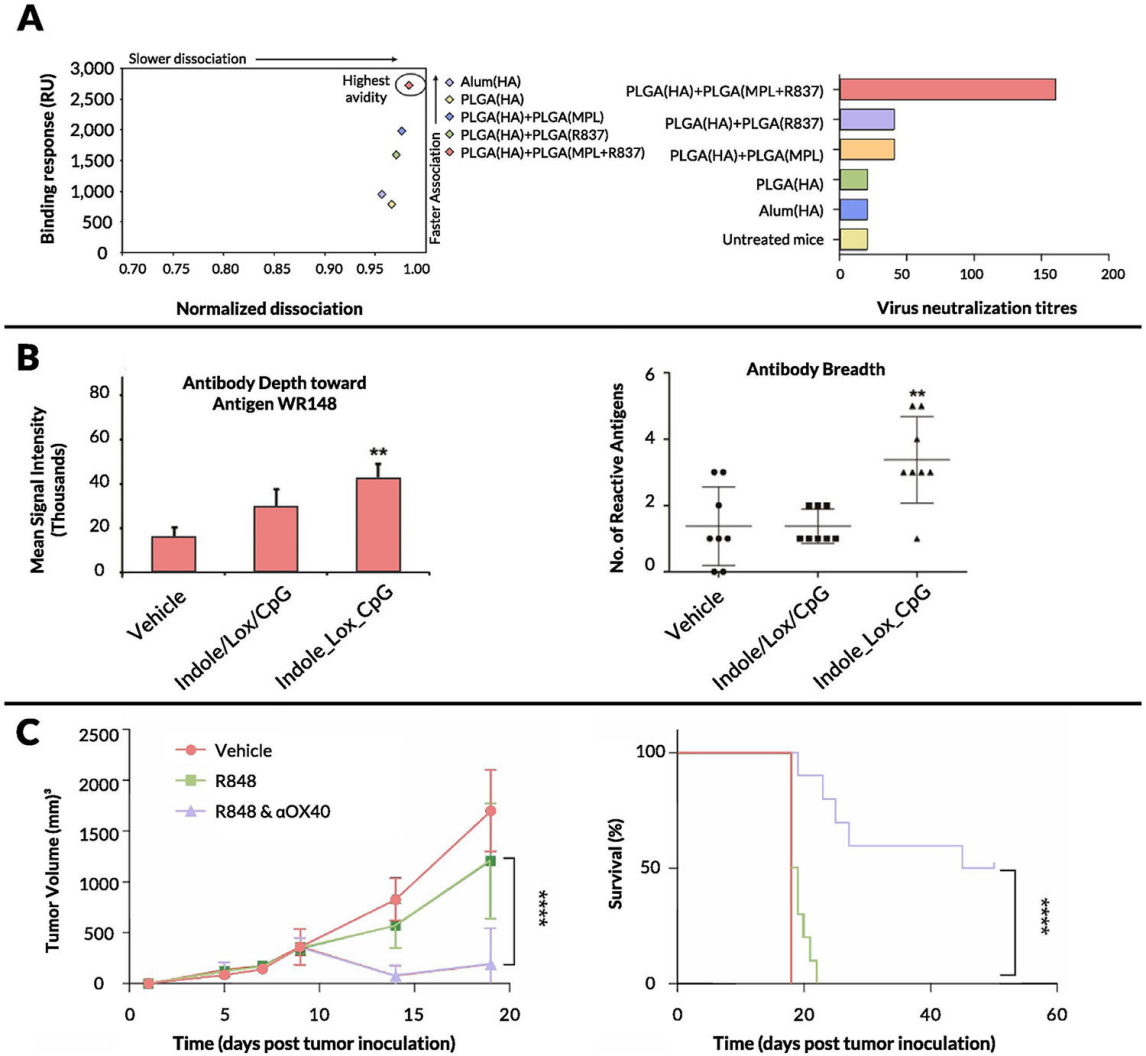
Combination delivery approaches resulting in synergic efficacy. (A) Combination of TLR7 agonist (R837) and TLR4 agonist (MPLA) encapsulated in PLGA particles synergistically improves antibody responses against H5N1-influenza-derived HA. (B) A single molecule containing TLR7 agonist (loxoribine), TLR4 agonist (pyrimido-indole) and TLR9 agonist (CpG ODN) improves antibody responses compared to the admixed formulation of the three agonists. (C) Combination of TLR7/8 agonist (R848) and anti-OX40 antibody demonstrates synergistic anti-tumor effects in A20 B cell lymphoma mouse model.

Drug carriers may have an important role to play in maximizing the impact of synergistic immune agonist drugs. Pulendran and coworkers utilized NPs similar to those described above in section 4.1.2. and demonstrated that R837 and monophosphoryl lipid A (a synthetic TLR4 agonist) encapsulated in PLGA NPs functioned synergistically when tested in multiple antigen models [316]. They observed a significant increase in antigen-specific neutralizing antibodies and enhanced persistence of germinal centers and plasma cell responses (Fig. 8A). These antibody responses were dependent on direct triggering of both TLRs on B cells and DCs, in addition to T cell help. The system was tested in other vaccination models of RMs and showed significantly improved levels of antigen-specific B cell responses as well as durable protection against rechallenge [317]. Toxicity issues—a key concern when combining therapies—limited the dose of the TLR agonists in these NPs [318]. This combination of TLR7/8 agonists with TLR4 agonists has been verified in numerous other studies [319-321]. For example, Carter and colleagues tested this combination encapsulated in anionic liposomes with a recombinant malaria antigen [319]. They observed increased levels of antigen-specific IFN-γ production and IgG_2_:IgG_1_ levels indicative of an improved Th1 response. Hook and colleagues looked beyond liposomal structures toward cubosomes (i.e., cubic phase liquid crystalline nanostructures formed in this case from lipids), achieving similar responses [320]. Additionally, Carson and colleagues observed rapid seroconversion, antigen sparing, and protective efficacy using their TLR4-TLR7 agonist combination [321]. Shattock and colleagues demonstrated dose- and administration route-dependence of the synergy between TLR4 and TLR7/8 agonists in a Gottingen minipig model [322].

Taking TLR combinations a step further, Haynes and colleagues added a TLR9 agonist into the mix and observed a durable antigen-specific antibody response [323]. Moreover, Roy and colleagues loaded this combination onto PLGA NPs and demonstrated synergistic activity in the context of antigen cross-presentation *in vitro* as well as lymph node germinal center and T helper cell responses *in vivo* [324]. Linking IMDs with other TLR agonists in a single molecule has also been shown to enhance activation of NF-κB and inflammatory cytokines *in vitro* and improved antibody response *in vivo* (Fig. 8B) [325]. Analysis of APC activation by multifunctional conjugates linking ligands for multiple distinct TLRs into the same molecule indicates that synergy between TLR agonists depends upon the physical length of the linker bridging innate immune ligands, the choice of ligand combination, and the dose [266,326]. These studies represent important early steps toward elucidating structure–activity dependence of TLR synergies, and complement work aiming to define rational TLR combinations based on the interplay between different intracellular signaling pathways [327-333].

In addition to studies of TLR agonist combinations as vaccine adjuvants, other groups have focused on the potential of TLR7/8 ligands to synergize with other immunotherapy modalities for cancer treatment. Illidge and colleagues combined radiation therapy with systemic R848 and observed improved antitumor efficacy in multiple tumor models [334]. Levy et al. demonstrated that the synergistic effect of TLR7/8 agonists with anti-OX40 antibodies was explained by the fact that TLR7/8 agonists such as R848 induce expression of the OX40 target on CD4 T cells in the tumor microenvironment (Fig. 8C) [335]. Although TLR7/8 agonists could be substituted by TLR9 agonists, surprisingly, checkpoint inhibitors against PD-1, PD-L1, or CTLA-4 could not substitute for anti-OX40 antibodies. A number of other studies, however, have reported synergy between TLR7/8 agonists and checkpoint inhibitors in various other murine tumor models [336-339]. Other attempts to augment the efficacy of synthetic TLR7/8 agonists have included combinations with antibodies such as anti-EGFR and anti-HER2/neu, cytokines such as IL-2, and photothermal therapies [340-345].

## 5. Conclusion and Future Outlook

Substantial evidence from preclinical studies and clinical trials suggests that synthetic TLR7/8 ligands have the potential to be powerful immunomodulators, vaccine adjuvants, and cancer therapeutics, but challenges in achieving suitable efficacy while avoiding toxicities remain a significant barrier. Recent elucidations of crystal structures, SAR analyses, and delivery approaches have led to steady progress, though systemic administration of TLR7/8 agonists still faces challenges [346]. Moreover, defining optimal dosing thresholds and timing intervals will be critical given that research on these compounds has followed closely on the heels of progress made on synthetic TLR4 and TLR9 agonists wherein significant efforts toward understanding tolerability and circadian effects have been pursued [347-350]. TLR tolerance, which is defined as a transient state of refractoriness of TLRs to subsequent activation post initial dosing, has evolved to avoid the induction of auto-immunity through repeated TLR agonism; however, it has important implications for dosing schedules involving synthetic TLR7/8 agonists that aim to generate pro-inflammatory immune responses [351]. Although TLR7 tolerance has been observed in multiple studies, the mechanism of induction is still an active area of research [352-356]. Initial studies provide confidence that dosing schemes can be devised that avoid TLR7 tolerance though this phenomenon must be studied in further detail to ensure maximum efficacy of these compounds [357,358]. Moreover, the dependence of TLR expression and activity of agonists on circadian biology has yielded promising results in other agonist systems but is yet to be explored in detail for TLR7/8 agonism [359,360]. Finally, as touched upon briefly in the previous sub-section, defining the synergistic role of synthetic TLR7/8 agonists in combination with established as well as emerging treatments is of utmost importance in our endeavor to achieve long-lasting cures to cancer, infectious diseases, and allergic and autoimmune conditions.

## Figures and Tables

**Table 1:** TLR7/8 expression levels and downstream effects

	Species	Sub-classification	TLR7/8 expression	Downstream effects (autocrine)	Response	References
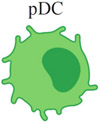	mouse	CD11c^−^ Ly6c^+^	7++ : 8−	type I IFNs	anti-viral state	[37] [39] [43] [50] [70]
	macaque	CD123^+^				
	human	CD123^+^				
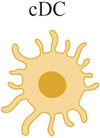	mouse	DN	7++ : 8++	pro-inflammatory cytokines and chemokines including TNF-α, IL-6, IL-12 and MCP-1	acute inflammation, maturation of DCs and expression of costimulatory molecules for priming T cells	[36] [37] [39] [44] [45]
		CD4^+^				
		CD8α^+^	7+/− : 8++			
	human	CD11c^+^x	7+ : 8+			
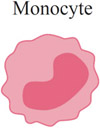	mouse	GR1^+^Ly6c^high^	7++ : 8++	activation of FcγR, differentiation into Mφ and DCs, secretion of pro-inflammatory cytokines such as IL-12	acute inflammation	[36] [37] [39] [52] [61]
	human	CD14^+^	7+/− : 8++			
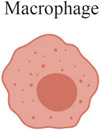	mouse	RAW264.7	7++ : 8++	pro-inflammatory cytokines including TNF-α and IL-12	M1 type polarization effects	[37] [39] [62]
		Bone-marrow derived macrophages				
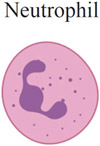	mouse	Ly6G^+^ CD11b^+^	7+ : 8?	including IL-8, release of reactive oxygen species	acute inflammation	[56] [57] [63] [64]
	human	CD62L^+^ CD16^+^	7+/− : 8++			
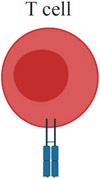	mouse	CD4^+^	7+: 8+*	pro-inflammatory cytokine secretion, suppression of T _reg_ activity	Proliferation and IL-2 production, Shift toward Th1 response	[53] [54]
	human					
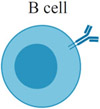	mouse	CD19^+^ or CD20^+^	7++ : 8−	increased proliferation, antibody secretion, cytokine production such as TNF-α and IL-6	humoral response	[69] [70] [72] [73] [74]
	macaque					
	human					

* a particular subset of CD4 T cells which also express CD25 referred to as regulatory T cells (Tregs) express TLR8

**Table 2:** List of synthetic TLR7/8 agonists in different stages of clinical development

Active	Drug Name	Company Name	Therapy Area	Development Stage	Route of Administration	Target
1	Imiquimod	3M Pharnaceuticals	Skin conditions, Infectious Diseases, Advanced Cancers	Marketed	Topical	TLR7
2	Resiquimod (R-848)	Galderma (Originally 3M Pharmaceuticals)	Skin conditions, Infectious Diseases, Advanced Cancers	Phase II	Topical, Oral, Intra-tumoral	TLR7/8
3	Motolimod (VTX-2337)	Bristol-Myers Squibb Co (previously VentiRx Pharmaceuticals)	Advanced Cancers	Phase II	Subcutaneous, Intra-tumoral	TLR8
4	Selgantolimod (GS-9688)	Gilead Sciences Inc	Infectious Diseases	Phase II	Oral	TLR8
5	NKTR-262	Nektar Therapeutics	Advanced Cancers	Phase II	Intra-tumoral	TLR7/8
6	RG-7854 (RO 7020531)	F. Hoffmann-La Roche Ltd	Infectious Diseases	Phase II	Oral	TLR7
7	DSP-0509	Sumitomo Dainippon Pharma Co Ltd	Advanced Cancers	Phase II	Intravenous	TLR7
8	BDB-001	Seven and Eight Biopharmaceuticals Corp	Advanced Cancers	Phase I	Intravenous	TLR7/8
9	BDC-1001	Bolt Biotherapeutics Inc	Advanced Cancers	Phase I	Intravenous	TLR7/8 (HER2+ cells)
10	LHC-165	Novartis AG	Advanced Cancers	Phase I	Intra-tumoral	TLR7
11	SHR-2150	Jiangsu Hengrui Medicine Co Ltd	Advanced Cancers	Phase I	Oral	TLR7
12	JNJ-4964 (TQ-A3334)	Johnson & Johnson, Chia Tai Tianqing Pharmaceutical Group Co Ltd	Infectious Diseases, Advanced Cancers	Phase I	Oral	Not specified
13	Vesatolimod (GS-9620)	Gilead Sciences Inc	Infectious Diseases	Phase I	Oral	TLR7
14	RO-7119929	F. Hoffmann-La Roche Ltd	Advanced Cancers	Phase I	Oral	TLR7
15	DN-1508052	Shanghai De Novo Pharmatech Co Ltd	Advanced Cancers	Phase I	Subcutaneous	TLR8
16	VTX-1463	Bristol-Myers Squibb Co (previously VentiRx Pharmaceuticals)	Respiratory Ailments	Phase I	Intra-nasal	TLR8
17	BNT-411 (SC1)	BioNTech SE	Advanced Cancers	IND/CTA Filed	Intravenous	TLR7
18	APR-003	Apros Therapeutics	Advanced Cancers	IND/CTA Filed	Oral	TLR7
Inactive/Discontinued						
1	Bropirimine	Pfizer Inc	Advanced Cancers	Discontinued/Inactive (Phase III)	Oral	TLR7
2	PF-4878691 (852-A)	Pfizer	Infectious Diseases, Advanced Cancers	Discontinued/Inactive (Phase II)	Intravenous, subcutaneous, Oral	TLR7
3	GSK-2245035	GlaxoSmithKline Plc	Respiratory Ailments	Discontinued/Inactive (Phase II)	Intra-nasal	TLR7
4	RG-7795 (ANA 773, RO 6864018)	F. Hoffmann-La Roche Ltd	Infectious Diseases, Advanced Cancers	Discontinued/Inactive (Phase II)	Oral	TLR7
5	Epitirimod (R-851)	Takeda (Originally 3M Pharnaceuticals)	Skin conditions	Discontinued/Inactive (Phase II)	Topical	TLR7
6	DSP-3025 (AZD-8848)	AstraZeneca Plc, Sumitomo Dainippon Pharma Co Ltd	Respiratory Ailments	Discontinued/Inactive (Phase II)	Intra-nasal	TLR7
7	Sotirimod (R-850, S-30594)	Meda AB	Skin conditions	Discontinued/Inactive (Phase II)	Topical	TLR7
8	Telratolimod (3M-052, MEDI-9197)	MedImmune, AstraZeneca Plc	Advanced Cancers	Discontinued/Inactive (Phase I)	Intra-tumoral	TLR7/8
9	Isatoribine (ANA-245)	F. Hoffmann-La Roche Ltd	Infectious Diseases	Discontinued/Inactive (Phase I)	Intravenous, Oral	TLR7
10	Loxoribine	Johnson & Johnson	Advanced Cancers	Discontinued/Inactive (Phase I)	intramuscular	TLR7
11	ANA-971	F. Hoffmann-La Roche Ltd	Infectious Diseases	Discontinued/Inactive (Phase I)	Oral	TLR7
12	ANA-975	Novartis AG	Infectious Diseases	Discontinued/Inactive (Phase I)	Oral	TLR7
13	RG-7863 (RO6870868)	F. Hoffmann-La Roche Ltd	Infectious Diseases	Discontinued/Inactive (Phase I)	Not specified	TLR7

**Table 3 :** List of synthetic TLR7/8 agonists at the pre-clinical stage

Drug Name	Formulation Approach	Company Name	Therapy Area	Route of Administration	Target
ALT-702	depot-forming peptide	Altimmune Inc	Advanced Cancers	Intra-tumoral	TLR7/8
GS-986	orally bioavailable small molecule	Gilead Sciences Inc	Infectious Diseases	Oral	TLR7
KUP-101	liposomal formulation	Kupando GmbH	Advanced Cancers	Intravenous	TLR4/7
PRTX-007	orally bioavailable small molecule	Primmune Therapeutics Inc	Infectious Diseases, Advanced Cancers	Oral	TLR7
PRX-034	orally bioavailable small molecule	Primmune Therapeutics Inc	Advanced Cancers	Oral	TLR7
S-34240	cream	Pfizer Inc (previously 3M Pharmaceuticals)	Skin Conditions	Topical	TLR7
TRANSCON	sustained release via cleavable linker	Ascendis Pharma	Advanced Cancers	Intra-tumoral	TLR7/8
SBT-6050	antibody-drug conjugate (IIER2)	Silverback Therapeutics Inc	Advanced Cancers	Intravenous	TLR8 (HER2+ cells)
SBT-6290	antibody-drug conjugate (Nektin4)	Silverback Therapeutics Inc	Advanced Cancers	Intravenous	TLR8
ZM-TLR8 agonist	small molecule with liver-targeting moiety	Zheming Biopharma	Infectious Diseases	Not specified	TLR8
VX-001	sustained release platform	Vaccex	Advanced Cancers	Intra-tumoral	TLR7/8
MBS-8	micellar formulation	MonTa Biosciences ApS	Advanced Cancers	Intravenous	TLR7
APR-002	small molecule with liver-targeting moiety	Apros Therapeutics	Infectious Diseases	Oral	TLR7
SNAPvax	self-assembling peptide nanoparticle	Avidea Technologies	Advanced Cancers	Intravenous	TLR7/8
R848-HA	hyaluronic acid (HA) hydrogel scaffold	STIMIT Technologies	Advanced Cancers	Intra-tumoral (intra-operative)	TLR7/8
